# Molecular principles of the assembly and construction of a carboxysome shell

**DOI:** 10.1126/sciadv.adr4227

**Published:** 2024-11-29

**Authors:** Peng Wang, Jianxun Li, Tianpei Li, Kang Li, Pei Cing Ng, Saimeng Wang, Vincent Chriscoli, Arnaud Basle, Jon Marles-Wright, Yu-Zhong Zhang, Lu-Ning Liu

**Affiliations:** ^1^ MOE Key Laboratory of Evolution and Marine Biodiversity, Frontiers Science Center for Deep Ocean Multispheres and Earth System & College of Marine Life Sciences, Ocean University of China, Qingdao 266003, China.; ^2^ Marine Biotechnology Research Center, State Key Laboratory of Microbial Technology, Shandong University, Qingdao 266237, China.; ^3^ Institute of Systems, Molecular and Integrative Biology, University of Liverpool, Liverpool L69 7ZB, UK.; ^4^ Biosciences Institute, Faculty of Medical Sciences, Newcastle University, Newcastle upon Tyne NE2 4HH, UK.

## Abstract

Intracellular compartmentalization enhances biological reactions, crucial for cellular function and survival. An example is the carboxysome, a bacterial microcompartment for CO_2_ fixation. The carboxysome uses a polyhedral protein shell made of hexamers, pentamers, and trimers to encapsulate Rubisco, increasing CO_2_ levels near Rubisco to enhance carboxylation. Despite their role in the global carbon cycle, the molecular mechanisms behind carboxysome shell assembly remain unclear. Here, we present a structural characterization of α-carboxysome shells generated from recombinant systems, which contain all shell proteins and the scaffolding protein CsoS2. Atomic-resolution cryo–electron microscopy of the shell assemblies, with a maximal size of 54 nm, unveil diverse assembly interfaces between shell proteins, detailed interactions of CsoS2 with shell proteins to drive shell assembly, and the formation of heterohexamers and heteropentamers by different shell protein paralogs, facilitating the assembly of larger empty shells. Our findings provide mechanistic insights into the construction principles of α-carboxysome shells and the role of CsoS2 in governing α-carboxysome assembly and functionality.

## Teaser

Insights into the mechanisms of α-carboxysome shell assembly can aid in carboxysome bioengineering.

## INTRODUCTION

Enhancing enzymatic and metabolic efficiencies is a key driving force for cellular organization to sustain life. This is prominently achieved through intracellular self-assembly and compartmentalization to physically and spatially sequester biological reactions (*1*). Increasing evidence has demonstrated that prokaryotic cells contain organelle-like structures, similar to those found in eukaryotic cells. These diverse organelle-like structures have various metabolic and physiological functions, facilitating adaptation to different environments and driving the evolution of cellular complexity (*2*).

Bacterial microcompartments (BMCs) are a versatile paradigm of protein-based organelles, which comprise a semi-permeable proteinaceous shell that encapsulates specific metabolic enzymes, playing essential roles in bacterial CO_2_ fixation, pathogenesis, and microbial ecology (*3*–*6*). The BMC shells selectively modulate the influx and efflux of metabolites, thus promoting enzymatic reactions within the compartment while sequestering toxic or volatile intermediates from the cytoplasm (*7*–*12*).

Among diverse BMCs, carboxysomes are specific CO_2_-fixing BMCs found in all cyanobacteria and many proteobacteria (*13*, *14*). The carboxysome uses a polyhedral protein shell to encapsulate the key CO_2_ fixation enzymes, ribulose-1,5-bisphosphate carboxylase/oxygenase (Rubisco) and carbonic anhydrase (CA) (*15*, *16*). The carboxysome shell permits the entry of HCO_3_^−^ and the Rubisco substrate, ribulose 1,5-bisphosphate (RuBP), while restricting the passage of CO_2_ and O_2_ (*17*, *18*). Furthermore, the encapsulated CA converts HCO_3_^−^ to CO_2_ under the regulation of RuBP (*19*). Overall, this intriguing natural organization of the carboxysome creates a high-CO_2_ microenvironment around Rubisco, thereby facilitating Rubisco carboxylation. This allows cyanobacteria and chemoautotrophs to play a substantial role in the global carbon cycle (*20*–*22*).

Carboxysome shells are typically composed of three groups of structural components that form hexamers, pentamers, and trimers (*14*, *23*). The self-assembly of these building blocks results in highly ordered icosahedral shells and cargo encapsulation to construct a functional carboxysome (*24*–*28*). However, the detailed protein-protein interactions that drive carboxysome shell assembly and the specific arrangement of building proteins within the shell have remained elusive.

Recent advancements in cryo–electron microscopy (cryo-EM) have revealed the internal Rubisco packaging and spatial organization within native α-carboxysomes (*15*, *29*, *30*). Given the organizational heterogeneity and the low density of the single-layer protein shell, the shell architectures in native carboxysomes are challenging to study. Recently, only the simplest and smallest, yet relatively uniform, α-carboxysome shell structure from *Prochlorococcus marinus* MED4 has been reconstructed at a resolution of 4.2 Å using a block-based reconstruction method (*31*). However, because of resolution limitations and averaging of small homogeneous blocks within the shell, the precise assignment of amino acid side chains that are responsible for direct interactions between shell components was not achievable.

Using *Escherichia coli* recombinant systems, it has been feasible to engineer intact α-carboxysomes (*26*, *32*, *33*), intact α-carboxysome shells (*34*–*37*), and simplified BMC shells (*16*, *38*–*40*). The recombinant systems allow for deliberate deletion or addition of components involved in shell assembly, permitting in-depth investigation of the functionality of specific components. Furthermore, high-resolution structures of shell variants in which specific components are removed or altered could provide sufficient fine-grained detail regarding the assembly and structural variability of α-carboxysome shells. Recent structural analysis of engineered mini-shells (composed of only CsoS1A hexamers and CsoS4A pentamers) has unveiled the basic principles of protein interactions that govern the construction of this simplified shell architecture. In particular, the essential role of the α-carboxysome linker protein, CsoS2, in bridging the shell and Rubisco cargos has been elucidated with molecular details (*16*, *29*, *41*). However, the protein interactions and organizational patterns in large shell structures with a full set of shell protein components remain largely unclear.

Here, we generated recombinant α-carboxysome shells of *Halothiobacillus neapolitanus* by expressing all the shell proteins (involving three CsoS1 hexameric proteins and two CsoS4 pentameric proteins), together with the C-terminal domain of CsoS2 (CsoS2-C) that mediates shell assembly. The resulting shell structures show various sizes, with a maximal size of 54 nm, representing the largest synthetic icosahedral BMC shells to date that are suitable for in-depth structural analysis. High-resolution cryo-EM structures of these “midi”-shell assemblies reveal diverse assembly interfaces formed between CsoS1 hexamers and key interacting sites between CsoS2-C and the shell proteins, which were previously unidentified in the mini-shells. These structural features are important for the formation of larger midi-shells. More intriguingly, we showed that the paralogs of CsoS1 hexamers and CsoS4 pentamers can form heterohexamers and heteropentamers, respectively, which may play important roles in governing α-carboxysome shell formation and function. Our findings provide molecular insights into the construction principles of large BMC shells and CsoS2-mediated α-carboxysome shell assembly, which could inform the design and reprogramming of shell structures with new biotechnological functions.

## RESULTS

### Assembly and structures of midi α-carboxysome shells

**Fig. 1.**
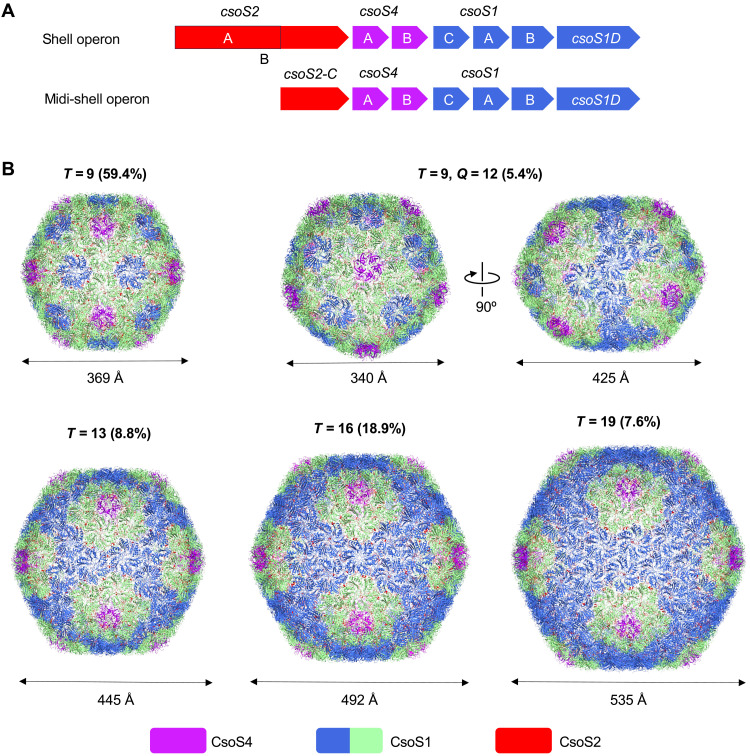
Design and overall cryo-EM structures of midi-shells. (**A**) Genetic organizations of shell operon and midi-shell operon. (**B**) Cryo-EM structures of different midi-shell forms of *T* = 9, *T* = 9 *Q* = 12, *T* = 13, *T* = 16, and *T* = 19, at the resolution of 2.3, 2.99, 2.93, 2.75, and 3.04 Å, respectively. The relative ratios and the diameters of different types of shells are indicated. The diameters of the shells are indicated. Shell components are colored purple (CsoS4 pentamer), blue/green (CsoS1 hexamer), and red (CsoS2). Hexamers at different relative positions are represented by different colors: green, shell hexamers adjacent to pentamers; blue, hexamers that are not adjacent to pentamers. The *T* and Q numbers are parameters for describing the geometry of polyhedral structures. *T* represents the area of a triangular facet within an icosahedral shell, whereas *Q* denotes the area of an elongated face within a prolate shell. Their respective computational formulas are as follows: *T* = *h*^2^ + *hk* + *k*^2^, *Q* = *hh*′ + *hk*′ + *kk*′. Here, *h* and *k* indicate the counts of two-step connections linking adjacent pentamers along the principal directions of the hexagonal lattice, respectively; *h*′ and *k*′ represent the counts of two-step connections linking diagonal pentamers within the prolate shell, respectively.

Previous studies demonstrated that expression of the *cso* genes encoding the whole set of shell proteins and CsoS2 (Fig. 1A, shell operon) results in the formation of α-carboxysome shells (~120 nm), which resemble the native shell structures with structural heterogeneity (*34*). In contrast, expression of minimal shell components CsoS1A and CsoS4A results in the formation of icosahedral mini-shells (*T* = 3, ~22 nm; *T* = 4, ~25 nm) (*16*, *38*). Coexpressing CsoS2 together with CsoS1A and CsoS4A generated larger mini-shells (*T* = 9, ~37 nm), in which the C terminus of CsoS2 (CsoS2-C) serves as a “molecular thread” to tether CsoS1A and CsoS4A, promoting mini-shell formation (*16*). This variation in the size of engineered shells allowed us to question what determines the α-carboxysome shell shape and size and how to manipulate them.

To address this question, we created a midi-shell construct to express the whole set of shell proteins and CsoS2-C (Fig. 1A). Expressing the midi-shell components resulted in the formation of larger icosahedral shells with the size ranging from 37 to 54 nm, notably larger than the mini-shells, while smaller than intact empty shells (fig. S1). Cryo-EM analysis revealed that the midi-shell construct produced shells that displayed various sizes and symmetries, including *T* = 9, *T* = 9 *Q* = 12, *T* = 13, *T* = 16, and *T* = 19 (Fig. 1B, fig. S2, and table S1), more intricate than mini-shells that only form the maximum of *T* = 9 shells. Among them, the *T* = 9 shell has the highest proportion, while the *T* = 9 *Q* = 12 shell has the lowest proportion (fig. S3). In addition to CsoS1A and CsoS4A involved in the mini-shells, the midi-shell construct expressed additional shell proteins, including CsoS4B, CsoS1B, CsoS1C, and CsoS1D, as well as CsoS2-C instead of full-length CsoS2 (Fig. 1A and table S2). Only the N-terminal domain (CsoS2-N) and middle region (CsoS2-M) of CsoS2 were absent in midi-shells, in contrast to entire empty shells produced by the *cso* operon (*34*). These results indicate the importance of additional shell components (CsoS4B, CsoS1B, CsoS1C, and CsoS1D), in addition to the recently studied CsoS2-M (*42*–*44*) and the yet-to-be-defined CsoS2-N, in shaping the α-carboxysome shell.

The cryo-EM structures of *T* = 9, *T* = 9 *Q* = 12, *T* = 13, *T* = 16, and *T* = 19 shell assemblies were determined at 2.3-, 2.99-, 2.93-, 2.75-, and 3.04-Å resolution, respectively (Fig. 1B and table S1). Consistent with the *T* = 9 mini-shell, all the shell assemblies produced from the midi-shell construct exhibit additional densities at the shell inner surfaces. These densities correspond to CsoS2-C (fig. S4). The resulting *T* = 9 shell atomic models closely resemble the *T* = 9 mini-shell, with root mean square deviations (RMSDs) of 0.33 Å over 928 Ca atoms. The *T* = 9 shell consists of 80 CsoS1 hexamers, 12 CsoS4 pentamers, and 60 CsoS2-C. The *T* = 9 *Q* = 12 shell has a dimension of 34.0 nm by 34.0 nm by 42.5 nm, comprising 95 CsoS1 hexamers, 12 CsoS4 pentamers, and 60 CsoS2-C. The *T* = 13 shell has a diameter of ~45 nm, composed of 120 CsoS1 hexamers, 12 CsoS4 pentamers, and 120 CsoS2-C. The *T* = 16 shell had a diameter of ~49 nm, consisting of 150 CsoS1 hexamers, 12 CsoS4 pentamers, and 120 CsoS2-C. The *T* = 19 shell (~54 nm, 14.8 MDa) is composed of 180 CsoS1 hexamers, 12 CsoS4 pentamers, and 120 CsoS2-C, with an asymmetric unit composed of three BMC-H (CsoS1A/B/C) hexamers and one BMC-P subunit (CsoS4A/B). It represents the largest recombinant icosahedral BMC shell with detailed structure determined at a high resolution, to our knowledge.

Note that the sequence-level differences among CsoS1A, CsoS1B, and CsoS1C are subtle (fig. S5A), posing a challenge to distinguish them structurally in the midi-shell reconstructions. CsoS1A and CsoS1C differ by only two amino acids (3rd and 97th amino acids). The local densities for these two amino acids are poor, making it difficult to distinguish CsoS1A and CsoS1C at the current resolution (fig. S5, A to D). To further verify this, we generated a *T* = 9 mini-shell in which CsoS1C is the only hexamer in the construct (fig. S6). The cryo-EM structure at 1.8-Å resolution showed that the resulting *T* = 9 shell resembles the *T* = 9 mini-shell formed by CsoS1A (*16*). CsoS1C in the mini-shell structure exhibited a similar structure to CsoS1A in the mini-shell and CsoS1C at the crystallized state [Protein Data Bank (PDB): 3H8Y]. CsoS1B is a low-abundance shell hexamer (fig. S1A) (*26*). Given the high level of sequence identity between CsoS1B and CsoS1A, it is challenging to differentiate these in cryo-EM reconstructions, even at high resolutions (fig. S5, A to D). Furthermore, the icosahedral averaging imposed on our *T* = 9 reconstruction would blur the additional C-terminal α helix in CsoS1B (see detailed discussion below). From the current density maps, shell pentamers were all assigned to CsoS4A, although CsoS4B was detected in the midi-shell samples via mass spectrometry (table S2). Similar to CsoS1A and CsoS1B, the signal from CsoS4B is likely to be averaged out in the icosahedral reconstructions with CsoS4A due to their structural similarity (fig. S5, E to G) and the low content of CsoS4B in α-carboxysome shells (see detailed discussions below) (*26*). Therefore, all structural models of the midi-shells were built using the sequences from CsoS1A and CsoS4A for hexamers and pentamers, respectively, which are highly conserved across α-cyanobacteria and some proteobacteria (*16*). In addition, no CsoS1D was identified in the midi-shell structures despite that it was detected in midi-shell samples by both SDS–polyacrylamide gel electrophoresis (SDS-PAGE) and mass spectrometry (fig. S6B and table S2). Consistently, attempts to structurally detect CsoS1D within mini-shells also failed, although it was detected in purified CsoS1C-formed mini-shells (fig. S6B), in agreement with previous findings (*38*). These results, along with our previous findings showing that CsoS1D has a low content in native α-carboxysomes (*26*), suggest that incorporation of CsoS1D into shells may be distinct from that of other BMC trimers that were found to be abundant in synthetic BMC shells (*39*, *45*).

### Protein organization and interaction interfaces in midi-shells

**Fig. 2.**
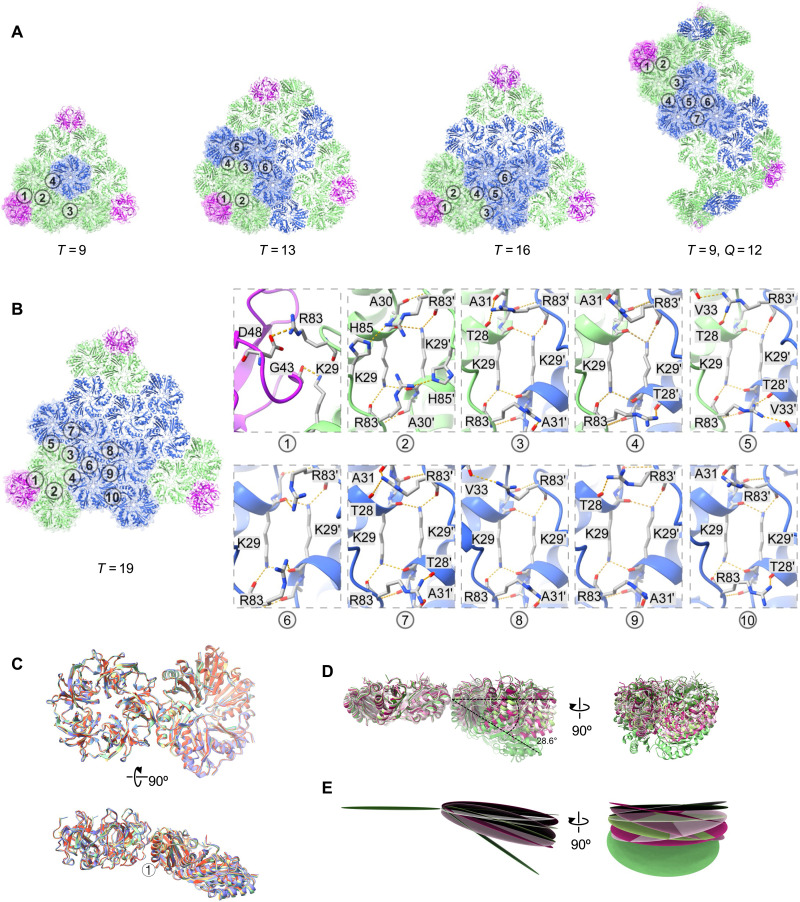
The shell proteins and interfaces in midi-shells. (**A**) Overall organization of *T* = 9, *T* = 13, *T* = 16, and *T* = 9 *Q* = 12 shells, with labeled assembly interfaces between capsomeres. (**B**) Details of interacting residues in the 10 different assembly interfaces of the *T* = 19 shell. (**C**) Overlay of interface 1 from *T* = 9 (yellow), *T* = 13 (green), *T* = 16 (blue), *T* = 19 (purple), and *T* = 9 *Q* = 12 (orange) shells. (**D**) Side view of the overlay interfaces 2 to 10 from the *T* = 19 shell. (**E**) Vertical and horizontal angle differences of hexamer-hexamer interfaces 2 to 10 from the *T* = 19 shell. The hexamers are simplified to averaged planes.

In the five engineered midi-shell products, the RMSDs for the basic assembly units (capsomeres), CsoS1A hexamer and CsoS4A pentamer, were in the range of 0.310 to 0.585 Å and 0.274 to 0.579 Å, respectively (fig. S7, A and B). The changes to the shells of different sizes are mostly evident in the variation of hexamer numbers in shell facets. The rise in hexamer numbers results in an increase in the quantity of intercapsomere assembly interfaces (Fig. 2, A and B, and fig. S8). The *T* = 9 shell has a minimum of four different interfaces, whereas the *T* = 19 shell has a maximum of 10 different interfaces.

To distinguish the angular variations between capsomeres in different interfaces, we defined the vertical angle of capsomeres as α-angle and the horizontal angle as β-angle (fig. S9). Although the quantities varied, intercapsomere assembly interfaces of each shell can be divided into two categories: pentamer-hexamer interfaces and hexamer-hexamer interfaces. Despite the varied sizes of shells, pentamer-hexamer interfaces are highly conserved, primarily relying on the salt bridge formed between CsoS4A Asp^48^ and CsoS1A Arg^83^, as well as hydrogen bonds between CsoS4A Gly^43^ main-chain oxygen and CsoS1A Lys^29^ side chain (Fig. 2, B and C, and fig. S8). The angles between capsomeres in these interfaces vary slightly among all shells (α-angle: 29.4° to 31.5°; β-angle: 0°) (Fig. 2, B and C, and table S3). In contrast, the angles between capsomeres of the hexamer-hexamer interfaces show notable variations (0° to 33.6°), which is closely related to the differing numbers of hexamers accommodated (Fig. 2, D and E; fig. S7C; and table S3), highlighting the structural plasticity of α-carboxysome shells. Despite large variations in the tilt angle, interactions at the interface between adjacent hexamers are maintained by a hydrogen bond network mediated by Lys^29^ and Arg^83^ (Fig. 2B). However, the conformations of Lys^29^ and Arg^83^ vary at different interfaces, leading to the variations in amino acids that form hydrogen bonds with Lys^29^ and Arg^83^ (table S4). The structural conservation of pentamer-hexamer interfaces and the variety of hexamer-hexamer interfaces indicate that the change in the shell size is more likely driven by the variations in hexamer-hexamer interactions than at pentamer-hexamer interfaces (see detailed discussions below).

### CsoS2 adopts diverse conformations for shell binding

**Fig. 3.**
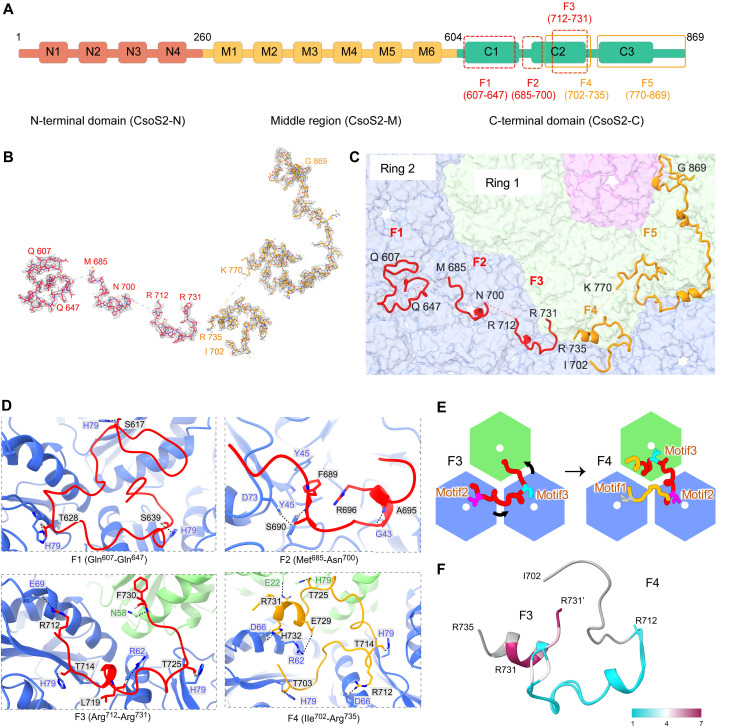
CsoS2 fragments and their interaction sites with shell proteins in midi-shells. (**A**) Domain arrangement of CsoS2. The N-terminal, middle and C-terminal domains are colored green, yellow, and pink, respectively. The four dashed boxes and one solid box indicate the fragments resolved in the *T* = 19 midi-shell. (**B**) Cryo-EM densities of newly identified CsoS2 F1 to F5 with atom models. (**C**) CsoS2 interactions with shell components, viewed from inside. CsoS4 pentamers are colored purple. Fragments belonging to two different CsoS2 chains are colored red and yellow, respectively. The first (hexamers adjacent to the pentamer) and second rings of CsoS1 hexamers are colored green and blue, respectively. (**D**) Interaction interfaces between CsoS2 F1 to F4 fragments and CsoS1A hexamers. (**E**) A pattern to indicate the relative position change of F3 and F4 on the shell inner surface. Residues of 712-731 in F3 and F4 are colored in red, and the others are in yellow. The residues of Thr^703^, Thr^714^, and Thr^725^ are marked in gray, magenta, and cyan, respectively. (**F**) Cartoon view of the structural alignment of F3 and F4 colored by Cα RMSDs values.

CsoS2 is a large polypeptide consisting of approximately 900 residues, comprising three distinct regions: CsoS2-N, CsoS2-M, and CsoS2-C (Fig. 3A) (*34*, *46*, *47*). Recent studies have revealed that CsoS2-N forms multivalent interactions with Rubisco, playing a pivotal role in facilitating the encapsulation and packaging of Rubisco (*29*, *41*). CsoS2-M and CsoS2-C attach to the inner surface of the α-carboxysome shell, functioning as a tether for cargo recruitment and mediating shell assembly through multivalent interactions with various shell components (*16*, *31*). Intriguingly, the key contacting regions of CsoS2-C with shell proteins were well structured, whereas other regions were intrinsically disordered, and high-resolution structural analysis of these regions has not been possible. The cryo-EM structures of *T* = 13, *T* = 16, and *T* = 19 midi-shells showed that the multivalent interactions between *Halothiobacillus* CsoS2 and shell proteins are present in all shell structures that are larger than the *T* = 9 shells (Fig. 3, B to D, and fig. S10), indicating a ubiquitous principle of the CsoS2-mediated shell assembly.

Notably, structural analysis of the large *T* = 19 shell revealed the highest number of CsoS2-C fragments and additional binding details. Most of the CsoS2-C was well-resolved in the cryo-EM map of the *T* = 19 shell, enabling precise assignment of amino acid residues of five CsoS2-C fragments in the *T* = 19 shell, namely, F1 (Gln^607^-Gln^647^), F2 (Met^685^-Asn^700^), F3 (Arg^712^-Arg^731^), F4 (Ile^702^-Arg^735^), and F5 (Lys^770^-Gly^869^) (Fig. 3, C and D, and fig. S10). The five fragments cover a substantial portion of entire CsoS2-C (Ser^604^-Gly^869^). In other midi-shells, the composition of structurally assigned CsoS2-C fragments varies according to the size of the shell (fig. S11). In the *T* = 16 shell, the identified CsoS2-C regions encompass almost identical F1 to F5 fragments as those of the *T* = 19 shell. In the *T* = 13 shell, only F3 (Arg^712^-Arg^731^), F4 (Arg^701^-His^732^), and F5 (Leu^773^-Gly^869^) were identified, whereas only F4 (Arg^712^-Arg^731^) and F5 (Leu^773^-Gly^869^) were determined in the *T* = 9 shell. These results imply that an increased number of interacting fragments leads to the formation of larger shells, highlighting the multivalent and elastic nature of CsoS2-mediated interactions involved in determining shell organization and shape. Together with our and others’ recent findings that addition of CsoS2-M leads to the formation of larger shells and carboxysomes (*42*–*44*), this implies that CsoS2 can be targeted as a means to recruit more cargo or to regulate carboxysome size.

The cryo-EM structures of the newly identified F1, F2, and F3 deviate from the predicted structures by AlphaFold2 (fig. S12). These fragments establish extensive hydrogen bonds and salt bridges with CsoS1A at distinct binding sites, featuring surface contact areas of 1849.9, 835.87, and 1205.9 Å^2^, respectively. Intriguingly, F1 and F3 contain three and two conserved repetitive Ile(Val)-Thr(Ser)-Gly ([IV][TS]G) motifs, which serve as common interaction motifs that interact with the β strand of CsoS1A through its His^79^ and hydrogen bonds in the main chain (Fig. 3D and fig. S13). This is in good agreement with the CsoS2-C fragments previously identified in the *T* = 9 mini-shell. At the interfaces where three CsoS1 hexamers form, these motifs of F1 and F3 individually interact with three CsoS1A hexamers, linking them together (Fig. 3, B to D). Moreover, unlike F1 that relies solely on [IV][TS]G motifs, F3 forms additional interactions with shell proteins, including hydrogen bonds between CsoS2 Leu^719^ and CsoS1A Arg^62^ and between CsoS2 Phe^730^ and CsoS1A Asn^58^, as well as a salt bridge between CsoS2 Arg^712^ and CsoS1A Glu^69^. In contrast, F2 lacks the [IV][TS]G motif (fig. S10A), and the binding of F2 to shell proteins does not rely on this motif; instead, it forms hydrogen bonds through the side chain and main chain of Ser^690^ with CsoS1 Asp^73^ and Tyr^45^ (Fig. 3D).

F4 and F5 align well with the three segments of CsoS2-C determined in the *T* = 9 mini-shell (Arg^712^-Arg^731^, Leu^773^-Gly^823^, and Glu^829^-Gly^869^) (fig. S13A) (*16*), suggesting analogous structural patterns. F4 demonstrates the structure of the 702-711 region and F5 connects the two previously separated fragments, Leu^773^-Gly^823^ and Glu^829^-Gly^869^ (fig. S13A).

Both F3 and F4 bind with the same CsoS1 hexamer in ring 1, which directly interacts with the pentamer at the vertex, and other two CsoS1 hexamers in ring 2, which are farther from the pentamer (Fig. 3C). F3 and F4 belong to the same region of CsoS2-C (Ile^702^-Arg^735^). Closer inspection revealed that F3 has a sequence overlap with F4 (Fig. 3A), although their conformations differ (Fig. 3, E and F). This region contains three repetitive [IV][TS]G motifs (motif1 to motif3, which are arranged based on amino acid sequences) (fig. S14). In F4, motif1 and motif2 interact with His^79^ on two hexamers of ring 2, whereas motif3 interacts with His^79^ on the hexamer of ring 1. In F3, motif2 and motif3 interact with His^79^ on two hexamers of ring 2, showing a notable difference from F4. Their binding sites are rotated clockwise by the distance of one motif (Fig. 3E). As documented above (Fig. 2E and fig. S7C), the angles of hexamer-hexamer interfaces vary. The conformational variations between F3 and F4 likely arise from the changes in the angles of interfaces between the three CsoS1 hexamers, one in ring 1 and two in ring 2 (Fig. 3, D and E).

On the basis of the structural trends resolved from the current density maps and the sequence overlap between F3 and F4 (Fig. 3F), it is speculated that F4 and F5 are parts of one CsoS2 polypeptide, whereas F1, F2, and F3 belong to another CsoS2, although we cannot rule out the possibility that F1, F2, F3, and F5 may belong to one CsoS2. Nevertheless, our results indicate that CsoS2-C adopts at least two distinct conformations for interacting with the shell. Intriguingly, the smaller *T* = 9 shells involved only one conformation of CsoS2-C, whereas the second conformation of CsoS2 appeared in the larger *T* = 13, *T* = 16, and *T* = 19 shells. In addition, the number of structurally assigned CsoS2-C fragments increased as the shell became larger (fig. S11). These observations indicate that the extent of CsoS2-C–mediated binding plays an important role in the formation of larger shells.

In the simple α-carboxysome shell structure from *Prochlorococcus* (PDB: 8WXB), two forms of CsoS2, one long and one short, were observed (fig. S15A) (*31*). Because of resolution limitations, both CsoS2 forms were suggested to share similar overall conformations. However, compared to their density map, our map shows a higher resolution, allowing for more precise assignments of amino acid side chains (fig. S15, B and C). Consequently, we identified two substantially different conformations of CsoS2 and analyzed how these two different conformations of CsoS2-C interact with the shell and the process by which these two conformations are formed.

### Formation of hetero-oligomers of CsoS1 and CsoS4 paralogs

The operon of the α-carboxysome shells of *Halothiobacillus* comprises three *csoS1* genes (*csoS1*A/B/C) and two *csoS4* genes (*csoS4*A/B) (Fig. 1A). The hexameric CsoS1 paralogs contain a single Pfam00936 domain and the CsoS4 pentamer-forming proteins contain a Pfam03319 domain. Removing the shell hexamers CsoS1A, CsoS1C, or CsoS1B has been suggested to result in a high CO_2_ requiring (HCR) phenotype; moreover, the deletion of CsoS1B appeared to have a lesser effect on the growth of the strain compared to the absence of CsoS1A or CsoS1C (*48*). Likewise, the absence of CsoS4A and CsoS4B also caused an HCR phenotype due to CO_2_ leakage (*49*). Why multiple paralogs of CsoS1 and CsoS4 are integrated into one α-carboxysome architecture remains enigmatic. Our midi-shell construct includes all the *csoS1* and *csoS4* genes; however, because of the limitations in structural analysis techniques and structural similarities of these paralogs, we could not directly distinguish the structures of CsoS1 paralogs, as well as those of CsoS4 paralogs, from the shell structures.

**Fig. 4.**
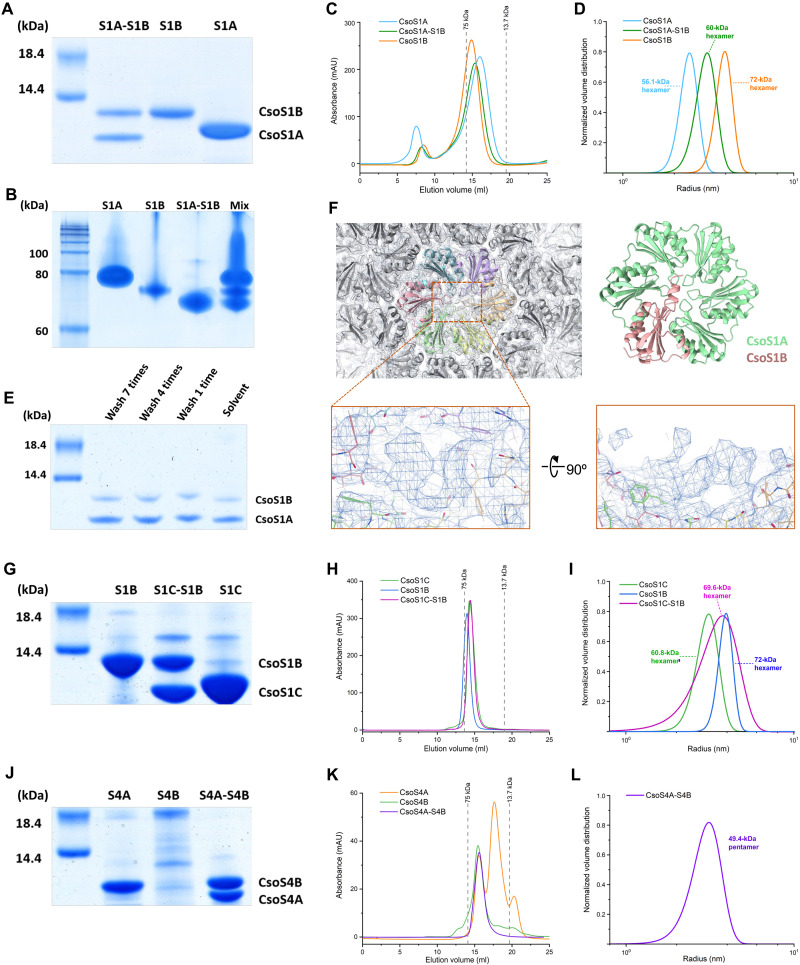
The hetero-oligomer formation between CsoS1 and CsoS4 Paralogs. (**A** and **B**) SDS-PAGE (A) and native-PAGE (B) analysis of individually expressed His-tagged CsoS1A (S1A), His-tagged CsoS1B (S1B), and coexpressed CsoS1A and His-tagged CsoS1B (S1A-S1B). The MIX lane sample in native-PAGE consists of a blend of individually purified proteins and coexpressed protein products. (**C** and **D**) Gel filtration chromatography (C) and DLS (D) results of His-tagged CsoS1A (blue), His-tagged CsoS1B (orange), and coexpressed CsoS1A and His-tagged CsoS1B (green). (**E**) SDS-PAGE analysis of crystals obtained from the co-expressed sample after various washing durations. (**F**) Extra density identified at the center and terminal regions of the CsoS1 hexamer in the C1 symmetry *T* = 16 shell map. (**G** to **I**) SDS-PAGE (G), gel filtration chromatographic (H), and DLS (I) analysis of CsoS1C (green), CsoS1B (blue), and coexpressed CsoS1C and CsoS1B (purple). (**J** and **K**) SDS-PAGE (J), gel filtration and chromatographic (K) analysis of CsoS4A (orange), CsoS4B (green), and coexpressed CsoS4A and CsoS4B (purple). (**L**) DLS analysis of coexpressed CsoS4A and CsoS4B (purple).

To evaluate this, we performed a detailed analysis of the assembly patterns of various CsoS1 and CsoS4 proteins. SDS-PAGE and immunoblot results revealed the integration of CsoS1B and CsoS1A/C in the purified midi-shells (fig. S1A). CsoS1A and CsoS1C cannot be distinguished by SDS-PAGE due to their similar molecular masses. To determine whether CsoS1B alone can form a hexamer, we heterologously expressed and purified CsoS1B using size exclusion chromatography. Nondenaturing PAGE showed that CsoS1B formed assemblies and dynamic light scattering (DLS) indicated that the molecular mass of the CsoS1B assemblies is ~72 kDa, confirming the ability of CsoS1B to form a hexamer (Fig. 4, A and B, and fig. S16). By generating and characterizing a CsoS4A-CsoS1B mini-shell construct, we found that CsoS1B as the sole shell hexamer cannot mediate the formation of mini-shells (fig. S17). This was also confirmed by previous studies (*38*). These findings demonstrated that solitary CsoS1B hexamers cannot independently mediate shell assembly.

Furthermore, we coexpressed CsoS1A and CsoS1B, where CsoS1B was His-tagged. Gel filtration chromatography and DLS data suggest that coexpression of CsoS1A and His-tagged CsoS1B yielded mostly hexamers, with the molecular mass falling between those of CsoS1A hexamers and CsoS1B hexamers (Fig. 4, C and D, and fig. S16). This indicates the formation of a heteromeric hexamer made by CsoS1A and CsoS1B, reminiscent of the CcmK paralogs in β-carboxysomes, which form CcmK3/K4 heterohexamers with a 1:2 stoichiometry (*50*, *51*). We then performed protein crystallization on the samples with coexpressed CsoS1A and CsoS1B. Good quality crystals were obtained, which diffracted to 2.1-Å resolution. A hexameric assembly was seen in this crystal structure (fig. S18 and table S5); however, we could not distinguish the CsoS1B subunits in the hexamer. To verify the loss of the CsoS1B signal caused by adding symmetry, we also attempted to process the data in P1 symmetry. However, because of defects in the crystal, no usable integration result was obtained. To determine whether the crystals obtained contained both proteins, samples were washed and subjected to SDS-PAGE analysis (Fig. 4E). The results confirmed the presence of both CsoS1A and CsoS1B proteins in the crystals. Regardless of the times of buffer washing, the ratio of CsoS1A and CsoS1B is ~3:1, equivalent to ~1.5 CsoS1B monomers per CsoS1A/B heterohexamer, which is consistent with the DLS results (Fig. 4D and fig. S16).

To visualize the existence of CsoS1A/B heterohexamers and prevent symmetry-caused biases in the process of differentiating CsoS1A and CsoS1B in the shells, we also analyzed the structures without introducing additional symmetries. Fortunately, the density maps for the *T* = 9 (C1) and *T* = 16 (C1) shells were retained at good resolution. For the *T* = 16 (C1) shell map, additional densities consistent with the CsoS1B C-terminal helix were observed at the central and terminal regions of the CsoS1 hexamer (Fig. 4F). Although building the structural model was challenging at this density, this result confirms that CsoS1A and CsoS1B can form heterohexamers during shell assembly. In contrast, the *T* = 9 (C1) shell map did not display any additional densities, suggesting that the CsoS1A/B heterohexamers are likely absent in the smaller *T* = 9 shells. Our mass spectrometry data further reveal that CsoS1B is present in significant quantities within purified midi-shells (table S2), which include the predominant *T* = 9 shells (59.4%) and less abundant *T* = 13 (8.8%), *T* = 16 (18.9%), and *T* = 19 (7.6%) shells. Collectively, these results imply that there is a correlation between the size/structure of the shell and the incorporation of CsoS1A/B heterohexamers and that CsoS1B is probably highly abundant in the larger shells. Nevertheless, we cannot exclude the possibility that the possible loss of CsoS1B signals in the *T* = 9 (C1) density map may be ascribed to particle superposition, instead of symmetry-related signal suppression.

The sequences of CsoS1A and CsoS1C differ by only two amino acids (fig. S5A), which do not affect the interfaces of the CsoS1 hexamer with neighboring shell proteins. Therefore, we speculate that CsoS1A and CsoS1C share similar features and can be naturally interchangeable during shell assembly. Consistently, with CsoS1C as the sole hexamer, a *T* = 9 mini-shell that structurally resembles the CsoS1A-containing *T* = 9 mini-shell (*16*) was obtained (fig. S6). We confirmed that, similar to CsoS1A, CsoS1C can form a hexamer alone, while when coexpressed with CsoS1B, CsoS1C can form CsoS1C/B heterohexamers (Fig. 4, G to I).

Similarly, while we were unable to detect the density for CsoS4B in midi-shells, coexpression of CsoS4A and CsoS4B led to formation of CsoS4A/B heteromeric pentamers (Fig. 4, J to L). Individual CsoS4A in solution exhibited a greater preference to form other aggregation forms in addition to pentamers (*52*) compared to CsoS4B pentamers or CsoS4A/B heteropentamers (Fig. 4K). We also attempted to obtain crystals of CsoS4A/B heterohexamers for further validation. However, no corresponding crystal was obtained.

Together, our results suggest that pairs of CsoS1 paralogs can form CsoS1A/B or CsoS1C/B heterohexamers and that the CsoS4 proteins can generate CsoS4A/B heteropentamers. The integration of these heterohexamers and heteropentamers in carboxysome shells, their actual percentage in the shell, as well as their functions in mediating shell assembly and permeability merit further investigations.

## DISCUSSION

In this study, we generated synthetic α-carboxysome midi-shells that closely mimic the protein composition of native α-carboxysome shells. High-resolution cryo-EM structures of these giant midi-shell assemblies, with a molecular mass of up to 14.8 MDa, allow us to examine, in molecular detail, the protein-protein interactions that drive the formation of the α-carboxysome shell as well as the interaction sites between the C-terminus of the linker protein CsoS2 and shell proteins, which play a crucial role in determining α-carboxysome shell assembly. In addition, we demonstrate the capabilities of CsoS1 and CsoS4 paralogs to form heterohexamers and heteropentamers, respectively. This enhances the variability of α-carboxysome shell formation and organization and is likely involved in modulating the shell permeability. These previously uncharacterized structural features provide insights into the molecular principles underlying CsoS2-mediated shell assembly and construction.

**Fig. 5.**
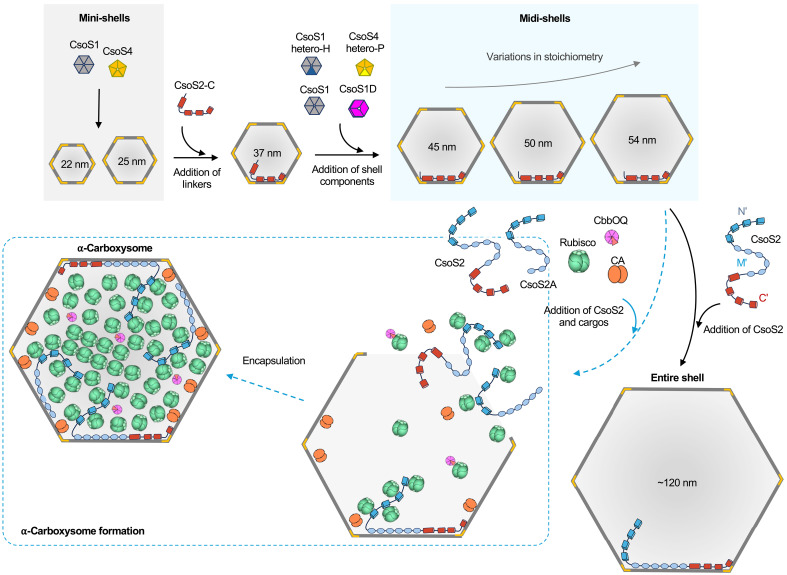
Proposed model of CsoS2-mediated assembly of α-carboxysome shells and intact α-carboxysomes. The combination of minimal shell components, consisting of a single type of hexamer and pentamer, can generate mini-shells with a maximal size of 25 nm. The presence of CsoS2-C, which stabilizes the interfaces between shell proteins, enables the expansion of mini-shells to 37 nm. Further incorporation of a full set of shell components, including homomultimers and heteromultimers, leads to formation of midi-shells with a maximal size of 54 nm. Replacing CsoS2-C with full-length CsoS2 yields large synthetic shells with an average size of ~120 nm, resembling native carboxysome shells. Furthermore, in the presence of CsoS2 and cargos in vivo, the shorter CsoS2 isoform (CsoS2A) assists the full-length CsoS2 isoform (CsoS2B) in recruiting Rubisco, along with the encapsulation of CA and the Rubisco activases CbbOQ, ultimately leading to the formation of an intact α-carboxysome (blue dashed arrows). Note that the numbers of carboxysome components do not represent the actual stoichiometry of α-carboxysome proteins.

On the basis of our findings and previous studies on the composition and assembly of α-carboxysomes and shells, we propose a model to illustrate the construction of shell assemblies and α-carboxysomes (Fig. 5). Mini-shells (~25 nm, *T* = 3) can be formed in the presence of a single type of hexamer (CsoS1A or CsoS1C) and pentamer (CsoS4A). The incorporation of CsoS2 or CsoS2-C can bridge the interfaces between adjacent shell components, resulting in the formation of larger mini-shell assemblies (~37 nm, *T* = 9) (*16*). Further integration of a full set of shell proteins, which naturally yields diverse homo-oligomers and hetero-oligomers, enables diverse interaction modes between CsoS2-C and hexamers, thereby facilitating the assembly of larger midi-shells (*T* = 13, *T* = 16, and *T* = 19) with a maximal size of 54 nm. This suggests that the presence of multiple hexamer and pentamer paralogs plays a role in determining the shell size. Although strictly, comparisons between different species are not valid, our results are consistent with the recent finding that *Prochlorococcus* α-carboxysome shell features only one type of CsoS1 hexamer compared to those from *Halothiobacillus* and many α-cyanobacteria and is smaller and more uniform compared to other α-carboxysome shells (*31*). A full set of shell proteins and full-length CsoS2 could form intact α-carboxysome shells of ~120 nm in diameter (*34*). This process is likely facilitated by the interactions between CsoS2-M and shell hexamers distant from the vertex pentamers, which promotes the stabilization of hexamer-hexamer interfaces and expansion of the shell size from 54 to 120 nm; this is supported by recent studies (*31*, *42*). Despite the important role of CsoS2-M in defining the shell size, CsoS2-M alone is insufficient to drive the formation of intact α-carboxysome shells and likely assists CsoS2-C in the assembly process (*43*, *44*). Furthermore, CsoS2A, the shorter CsoS2 isoform that lacks CsoS2-C, collaborates with the full-length CsoS2 isoform (CsoS2B) to recruit Rubisco using CsoS2-N (*29*, *41*). This process, along with the incorporation of CA and the CbbOQ Rubisco activase complex (*26*, *33*, *53*), eventually leads to the formation of an intact α-carboxysome. Overall, several factors are crucial for determining the diameter of the α-carboxysome shell, including shell protein composition and the incorporation and isoforms of CsoS2, in addition to cargo encapsulation.

This model also suggests that the biogenesis of α-carboxysomes likely adopts a concomitant and/or independent mode for shell formation and cargo assembly. This biogenesis pathway is supported by the observations that empty α-carboxysome shells could be reconstructed in *E. coli* (*34*), liquid-like Rubisco condensates could be generated with the assistance of CsoS2 (*29*, *41*), and partial α-carboxysome structures composed of incomplete shell assemblies and attached core proteins were visualized (*54*, *55*). It is notably distinct from the “core-first” mode of de novo β-carboxysome biogenesis (*24*, *56*) but reminiscent of the assembly pathway of propanediol-utilization microcompartments (*57*). Additional structural and biochemical details are required to elucidate de novo α-carboxysome biogenesis.

Given their self-assembly features and pivotal role in the global carbon cycle, carboxysomes have become increasingly important for fundamental research and bioengineering applications, with the intent of sustainably enhancing carbon fixation and catalytic performance. Compared to the mini-shells developed in our previous work (*16*), the larger midi-shells generated in this study offer substantially more internal space and surface area. This increased loading capacity allows for the immobilization of a higher number of cargo enzymes or molecules, significantly enhancing their potential as advanced nanoreactors and scaffolding/delivery systems for biotechnological applications. Moreover, the simple mini-shells are composed of only one type of shell hexamer and pentamer. In contrast, the midi-shells incorporate a complete set of shell components, ensuring that their selective permeability characteristics closely mimic those of complete shell structures. An in-depth understanding of the mechanisms underlying the assembly and heterologous reconstruction of carboxysomes will inform rational design and engineering of carboxysome-based nanostructures and nanomaterials with specific sizes, composition, and permeability for various biotechnological applications.

## MATERIALS AND METHODS

### Generation of constructs

All DNA assemblies between genes and linearized vectors were achieved by Gibson assembly (Gibson assembly kit, New England BioLabs, UK). Primers (table S6) were ordered from Integrated DNA Technologies. The midi-shell operon was generated by deleting the nucleotide sequence encoding the N-terminal and middle region of CsoS2 from the synthetic *cso*-2 operon derived from *H. neapolitanus* c2 (GenBank accession number: CP001801.1). Subsequently, the CsoS1C-based mini-shell operon was generated by deleting the genes encoding CsoS1A and CsoS1B from the midi-shell operon. Genes encoding CsoS4A and CsoS1B were amplified from the midi-shell operon and cloned into the pBAD vector linearized by Nco I and Eco RI to generate the *csoS4A*-*csoS1B* operon. All shell operons were then transformed to *E. coli* BW25113 cells (GenBank accession number: CP009273) for shell expression. For individual expression of the shell proteins, gene fragments encoding single shell proteins were amplified by polymerase chain reaction (PCR) using the *cso*-2 operon (*34*) as a template and then inserted into the pET-22b vector with a His tag fused at the C-terminal of the target gene. For coexpression, pairs of genes of different combinations (*csoS1A*-*csoS1B*, *csoS1C*-*csoS1B*, and *csoS4A*-*csoS4B*) were amplified and inserted into the pET-22b vector with the His tag fused at the C-terminal of CsoS1B or CsoS4B. All these constructs were verified by PCR and DNA sequencing.

### Isolation of α-carboxysome midi-shells

*E. coli* BW25113 cells containing the midi-shell construct were cultured in 2× YT medium supplemented with ampicillin at 37°C to an optical diameter of 600 nm of 0.6 to 0.8. After induction with 1 mM l-arabinose at 18°C for 16 to 18 hours, cells were collected and disrupted by high-pressure in TEMB buffer [10 mM tris-HCl, 1 mM EDTA, 10 mM MgCl_2_, and 20 mM NaHCO_3_ (pH 8.0)] containing 0.1% Protease Inhibitor Cocktail (Selleck) and 10% (v/v) CelLytic B Cell lysis Reagent (Sigma-Aldrich). Cell lysates were centrifuged at 50,000*g* for 30 min to clarify the supernatant. The clarified supernatant was then applied on the top of 5 ml of prechilled 30% (w/v) sucrose and centrifuged at 256,800*g*, 4°C for 16 hours. After centrifugation, pellets were resuspended with 2.5 ml of TEMB buffer and spun at 14,000*g* for 2 min to remove residual precipitate. The suspension was loaded onto a 10 ml of step sucrose gradient consisting of 10, 20, 30, 40, and 50% (w/v) sucrose fractions and was centrifuged at 70,000*g*, 4°C for 16 hours. The midi-shells were further purified using HiTrap Capto Q ImpRes anion exchange chromatography (Cytiva Life Sciences). Samples were dialyzed into buffer A (TEMB buffer with 50 mM NaCl) and eluted with continuous gradient of 0 to 100% buffer B (TEMB buffer with 1 M NaCl) over 27 column volumes. Fractions were collected and analyzed by SDS-PAGE. The fractions enriched with midi-shells were concentrated and loaded onto Superose 6 Prep grade column (Cytiva Life Sciences), following elution with buffer A. The eluted midi-shells were stored at 4°C for further analysis.

### SDS-PAGE, native-PAGE, and immunoblot analysis

Protein samples were mixed with SDS-PAGE loading buffer and electrophoresed in 15% SDS-PAGE gel after heating at 100°C for 15 min. For native-PAGE, native-PAGE loading buffer and 15% gel were used instead with nonheating to samples. Gels were stained with Coomassie Brilliant Blue for analysis. Immunoblot analysis was performed using primary rabbit polyclonal anti-CsoS1 (dilution, 1:3000; Agrisera, catalog no. AS142760), rabbit anti-CsoS2-C (dilution, 1:5000; synthesized by GenScript, NJ, USA), and horseradish peroxidase–conjugated goat anti-rabbit IgG secondary antibody (dilution, 1:10,000; Agrisera, catalog no. AS09602). Signals were visualized by using a chemiluminescence kit (Bio-Rad).

### Mass spectrometry analysis

The purified midi-shell samples were washed with PBS buffer and were treated for mass spectrometry analysis. Data-dependent liquid chromatography tandem mass spectrometry analysis was performed on a QExactive quadrupole-Orbitrap mass spectrometer coupled to a Dionex Ultimate 3000 RSLC nano-liquid chromatograph (Thermo Fisher Scientific, UK) installed with Xcalibur software version 4.1. A Mascot Generic File, created by Progenesis QI (version 3.0, Nonlinear Dynamics, Newcastle upon Tyne, UK), was searched against the *Halothiobacillus* carboxysome protein database from UniProt.

### Negative-staining transmission electron microscopy

Thin-section transmission electron microscopy (EM) was performed to visualize mini-shell and midi-shells isolated from *E. coli* strains. Isolated shell structures were characterized using negative staining EM. Thirty images were recorded using an FEI Tecnai G2 Spirit BioTWIN transmission electron microscope equipped with a Gatan Rio 16 camera. Image analysis was carried out by using ImageJ software. The midi-shell diameter data were randomly collected from 100 shell particles on EM images. The diameter of each polyhedral shell particle was measured by drawing diagonals three times from various angles, all intersecting at the same center point, using ImageJ software, and the resulting measurements were then averaged.

### Cryo-EM data collection

An aliquot (4 μl) of midi-shell samples was applied to a freshly glow-discharged holey carbon grid (Quantifoil Au R2/1, 200 mesh) with continuous carbon support. The grid was blotted for 2 s at 100% humidity at 10°C with a force level of −1 and immediately plunged into liquid ethane cooled by liquid nitrogen with Vitrobot Mark IV (Thermo Fisher Scientific, USA). The grids were loaded into a 300 kV Titan Krios G3i microscope (Thermo Fisher Scientific) equipped with a K3 BioQuantum direct electron detector (Gatan, USA) for data acquisition. A total of 4998 movie stacks were automatically recorded using EPU cryo-EM data acquisition software (Thermo Fisher Scientific) (*58*) at a total dose for a stack of 50 e^−^ Å^−2^ in a defocus range of −1.2 to −2.5 μm. A super-resolution mode was used at a nominal magnification of ×81,000 corresponding to a pixel size of 0.53 Å with the energy filter slit set to 20 eV.

For the CsoS1C-based mini-shell, 3 μl of purified samples (1.0 and 0.7 mg ml^−1^) were applied to glow-discharged grids (Quantifoil 1.2/1.3 200 mesh Cu grids). The grids were incubated at 4°C with 100% relative humidity for 0.5 s, blotted for 3 s, and then plunge-cooled in liquid ethane. Grids were screened with a Thermo Scientific Glacios at an accelerating voltage of 200 kV equipped with a Falcon-IV counting direct electron detector at ×240,000 magnification. For data acquisition, the grids were loaded onto a 300 kV Titan Krios K3 microscope equipped with a Gatan K3 direct electron detector. A total of 19,986 movie stacks were collected with EPU at a total dose for a stack of 46.1 e^−^ Å^−2^ in a defocus range of −0.6 to −2.0 μm. The nominal magnification was ×105,000 corresponding to a pixel size of 0.829 Å, with the energy filter slit set to 20 eV.

### Cryo-EM data processing

All movie stacks were corrected by MotionCor2.1 (*59*) with dose weighting. Contrast transfer function (CTF) parameters for each movie were estimated by CTFFIND-4 (*60*). Image processing was mainly performed using cryoSPARC 3.1.1 (*61*). After automatic particle picking and reference-free 2D classifications, 92,101 particles were selected, with obvious junk excluded from the particle set. The particles were 3D classified into six classes. After 3D nonuniform refinement and sharpening, global (per-group) CTF refinement and local (per-particle) CTF refinement were performed. The resolution was estimated on the basis of the gold-standard Fourier shell correlation at 0.143. The local resolution of the cryo-EM density map was generated using ResMap (*62*).

For the CsoS1C-based mini-shell, all data processing was performed in cryoSPARC v4.4.1. Patch-based motion correction and CTF correction were performed in cryoSPARC, and initial particle picking was performed with blob picker on a subset of micrographs. Initial reference-free 2D classes were generated and used for template-based picking on the full set of micrographs. Rounds of 2D classification and curation were performed on the fully extracted particle set to remove junk particles. An ab initio reconstruction job was performed with five classes corresponding to different assemblies of the mini-shell. The initial reconstruction and particle set corresponding to the *T* = 9 mini shell size were taken forward for heterogeneous refinement, followed by global and local CTF refinement jobs. Reference-based motion correction and Ewald sphere refinement were performed as the final polishing step. The final resolution was estimated on the basis of the gold-standard Fourier shell correlation at 0.143.

### Shell model building and refinement

For the *T* = 9 mini-shell and the *T* = 9 CsoS1C mini-shell, the structure of the *T* = 9 mini-shell (PDB: 8B12) (*16*) was utilized for initial model construction. Chimera (v1.17) (*63*) was used for rigid-body fitting. For the larger shells (*T* = 9 *Q* = 12, *T* = 13, *T* = 16, and *T* = 19), additional capsomeres were incorporated on the basis of the crystal structures of hexamers (PDB: 2EWH) (*64*) and pentamers (PDB: 2RCF) (*52*). The newly identified CsoS2 beyond the *T* = 9 mini-shell (PDB: 8B12) was manually traced into the density map using Coot (v0.9.4) (*65*). Further refinement of single asymmetric units of the icosahedral midi-shells was performed by Phenix (v1.20.1) (*66*) and Coot (v0.9.4) (*65*). The icosahedral models were reconstructed in Chimera (v1.17) (*63*). Structural alignment and analysis were conducted in ChimeraX (v1.16) (*67*) and Pymol (PyMOL Molecular Graphics System, version 2.0, Schrödinger, LLC). The quality of the final models is summarized in table S1.

### Expression and purification of CsoS1A/B/C and CsoS4A/B

Plasmids were transformed into *E. coli* BL21 (DE3) (Vazyme, China) cells. After induced by 0.3 mM isopropyl-β-d-thiogalactopyranoside at 18°C for 16 hours, cells were collected and disrupted by high-pressure in the lysis buffer [50 mM tris-HCl, 100 mM NaCl, and 5% glycerol (pH 8.0)]. The proteins were purified first with Ni^2+^-nitrilotriacetic acid resin (QIAGEN) and then fractionated by gel filtration on a Superdex 200 column (GE Healthcare) in the buffer containing 50 mM tris-HCl (pH 8.0) and 100 mM NaCl.

### DLS analysis

To measure the polymeric form and size distribution of individually and coexpressed shell proteins, 20 μl of purified samples was analyzed by using DynaPro NanoStar (Wyatt Technology, USA). All DLS measurements were done in triplicate and analyzed using Origin 2018 software.

### Crystallization and data collection

The purified coexpressed samples (CsoS1A-CsoS1B and CsoS4A-CsoS4B) were concentrated to approximately 5 mg ml^−1^ in 50 mM tris-HCl (pH 8.0) containing 100 mM NaCl and were crystallized at 18°C by the hanging drop method. The crystals of the CsoS1A-CsoS1B sample were obtained in a buffer containing 0.2 M lithium citrate tribasic tetrahydrate and 20% (w/v) polyethylene glycol, molecular weight 3350. X-ray diffraction data were collected on the Beamline BL02U1 at the Shanghai Synchrotron Radiation Facility using the detector DECTRIS EIGER2 S 9M. The initial diffraction dataset was processed using the x-ray detector software (*68*). The statistics of the data collection are shown in table S5.

### Crystal structure determination and refinement

The phases were determined by molecular replacement using the EPMR (evolutionary programming for molecular replacement) program and the CCP4 program Phaser (*69*). CsoS1A hexamer (PDB: 2EWH) (*64*) was used as the search mode. The structure refinement was performed using Coot (v0.9.4) (*65*) and Phenix (v1.20.1) (*66*). The quality of the final model is summarized in table S5.

### Alphafold2 structure prediction

The predicted structures were generated by AlphaFold2 (*70*) implementation in the ColabFold notebooks running on Google Colaboratory (*71*).
