# Supplementary material for: Molecular principles of the assembly and construction of a carboxysome shell
Source: Sci Adv. 2024 Nov 29;10(48):eadr4227. doi: 10.1126/sciadv.adr4227 (PMC11606499; doi:10.1126/sciadv.adr4227)
Supplement: Supplementary file 1 — Figs. S1 to S18 Tables S1 to S6 [file sciadv.adr4227_sm.pdf]

Science Advances
AAAS

Supplementary Materials for

Molecular principles of the assembly and construction of a carboxysome shell

Peng Wang *et al.*

Corresponding author: Yu-Zhong Zhang, zhangyz@sdu.edu.cn; Lu-Ning Liu, luning.liu@liverpool.ac.uk

Sci. Adv. **10**, eadr4227 (2024)
DOI: 10.1126/sciadv.adr4227

This PDF file includes:

Figs. S1 to S18
Tables S1 to S6

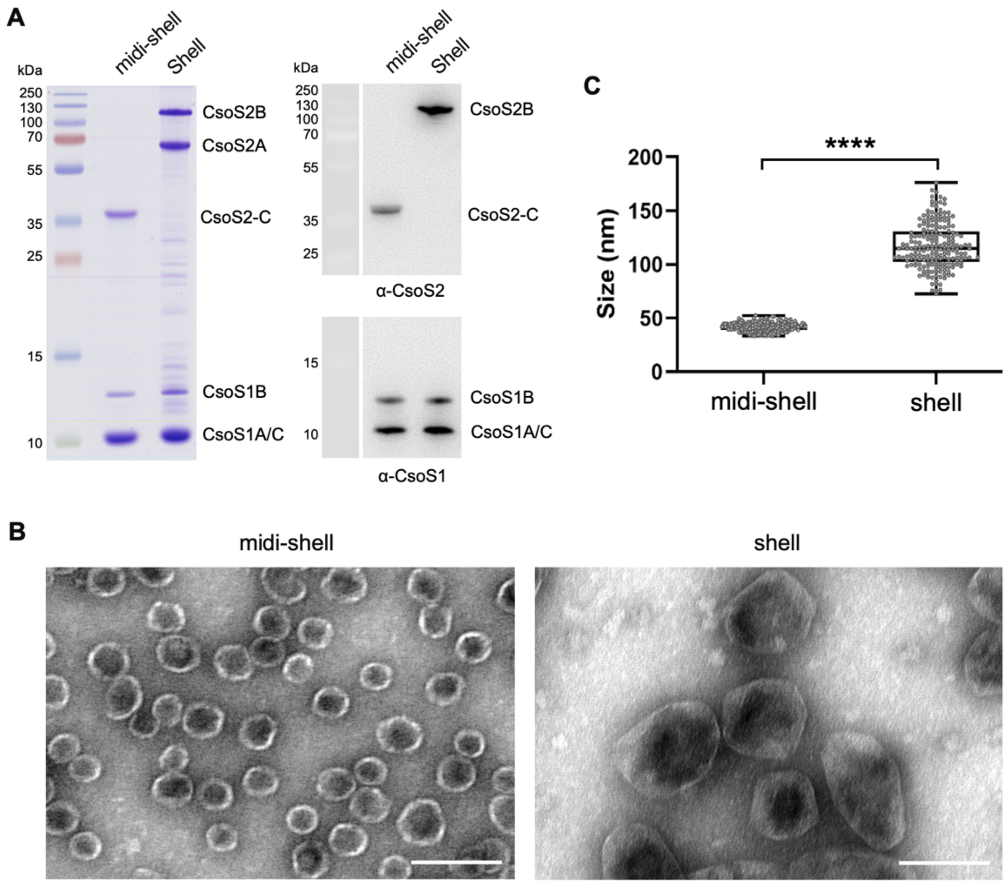

Supplementary Figure 1. Characterization of midi-shells. (A) SDS-PAGE (left) and immunoblot analysis (right) of purified shell and midi-shell. **(B)** Transmission EM of purified midi-shells and intact empty shells. Scale bar, 100 nm. **(C)** Size comparison of midi-shells and intact shells. ****$p <$ 0.0001 ($n = 100$, two-tailed unpaired t-test), implying the role of CsoS2 in confining shell architecture. Box plots indicate the median (middle line in the box), 25th percentile (bottom line of the box), and 75th percentile (top line of the box).

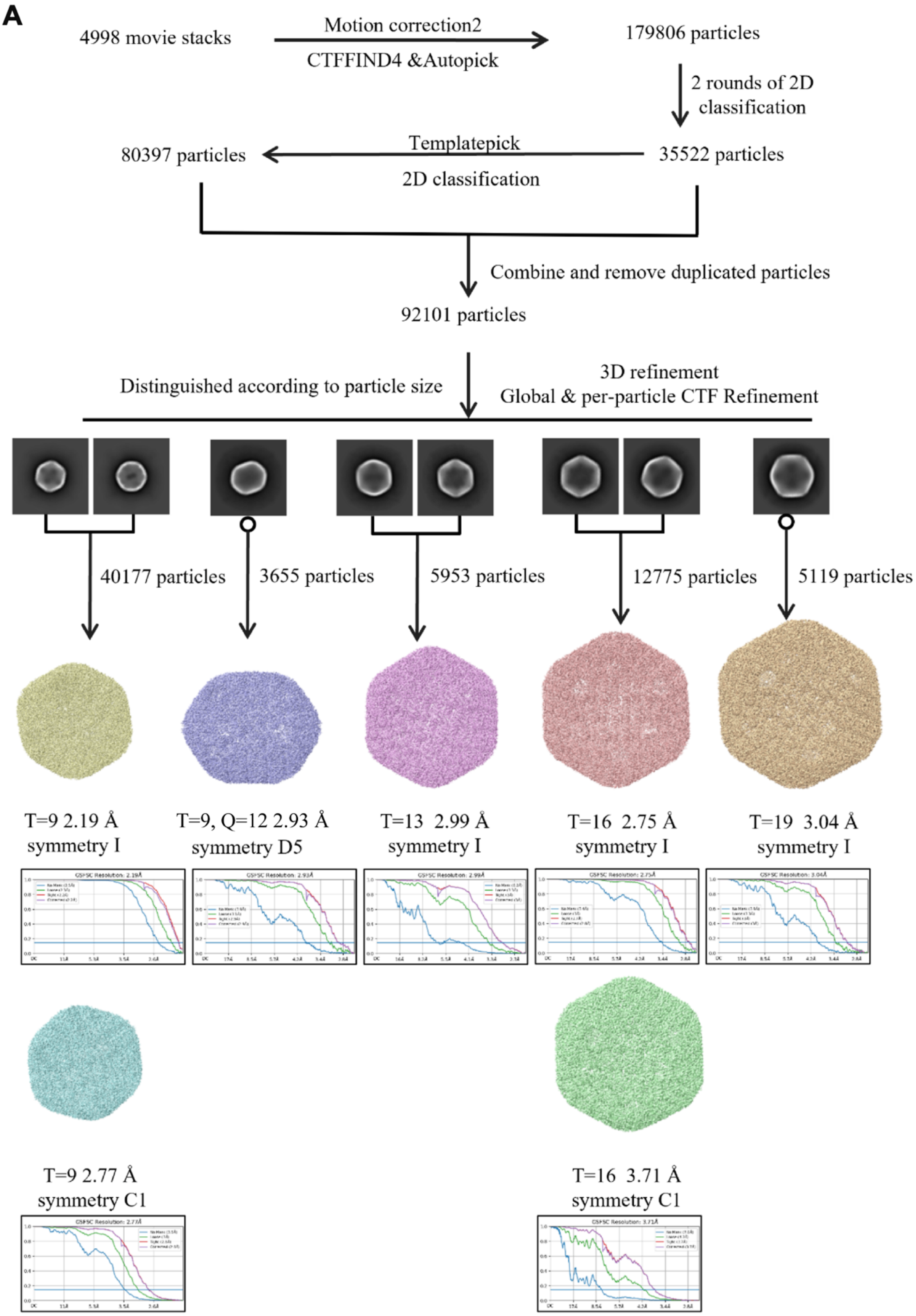
A
4998 movie stacks
Motion correction2
CTFFIND4 &Autopick
179806 particles
2 rounds of 2D classification
35522 particles
Templatepick
2D classification
80397 particles
Combine and remove duplicated particles
92101 particles
Distinguished according to particle size
3D refinement
Global & per-particle CTF Refinement
40177 particles
3655 particles
5953 particles
12775 particles
5119 particles
T=9 2.19 Å
symmetry I
T=9, Q=12 2.93 Å
symmetry D5
T=13 2.99 Å
symmetry I
T=16 2.75 Å
symmetry I
T=19 3.04 Å
symmetry I
T=9 2.77 Å
symmetry C1
T=16 3.71 Å
symmetry C1

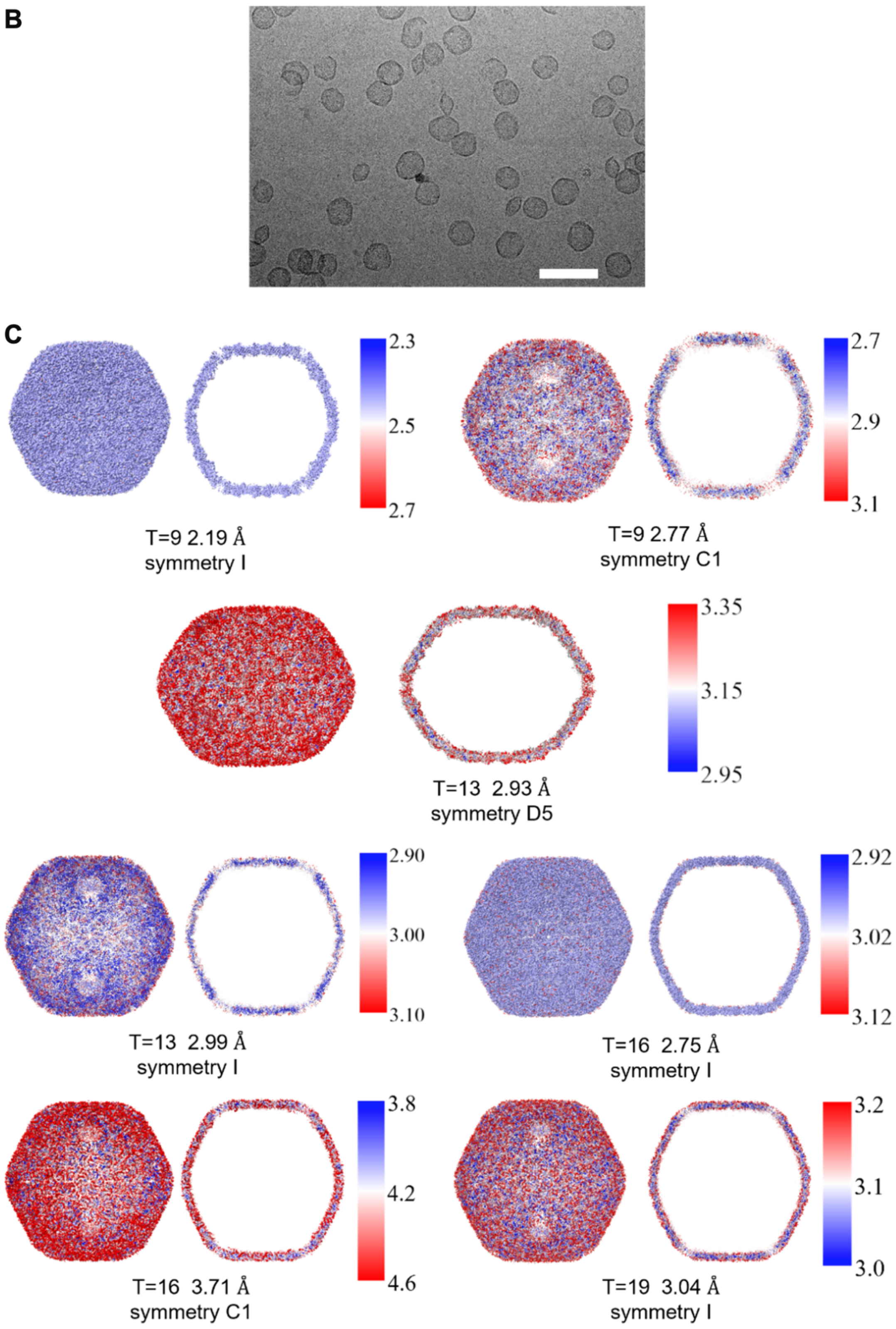

Supplementary Figure 2. Cryo-EM data processing for midi-shells. (A) Schematic flowchart for the cryo-EM data processing. The box size of representative 2D classes is 848 Å. **(B)** A representative cryo-EM micrograph of the midi-shells. Scale bar, 100 nm. **(C)** Local resolution distributions of the cryo-EM maps are shown in the top view (left) and central slice (right).

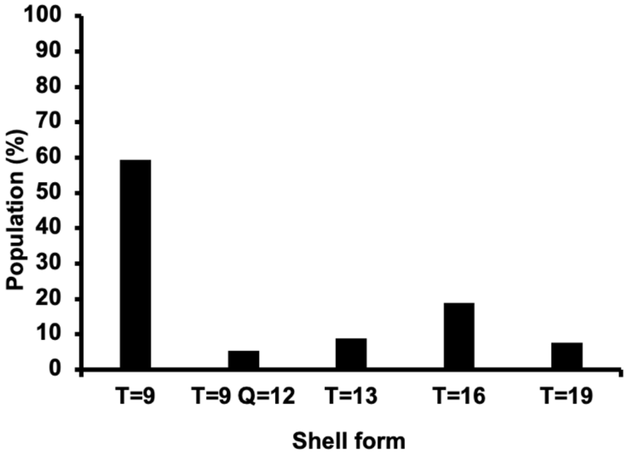

Supplementary Figure 3. Distribution of shell forms assembled with the midi-shell construct. *T*=9, 40,177 particles; *T*=9 *Q*=12, 3,655 particles; *T*=13, 5,953 particles; *T*=16, 12,775 particles; *T*=19, 5,119 particles.

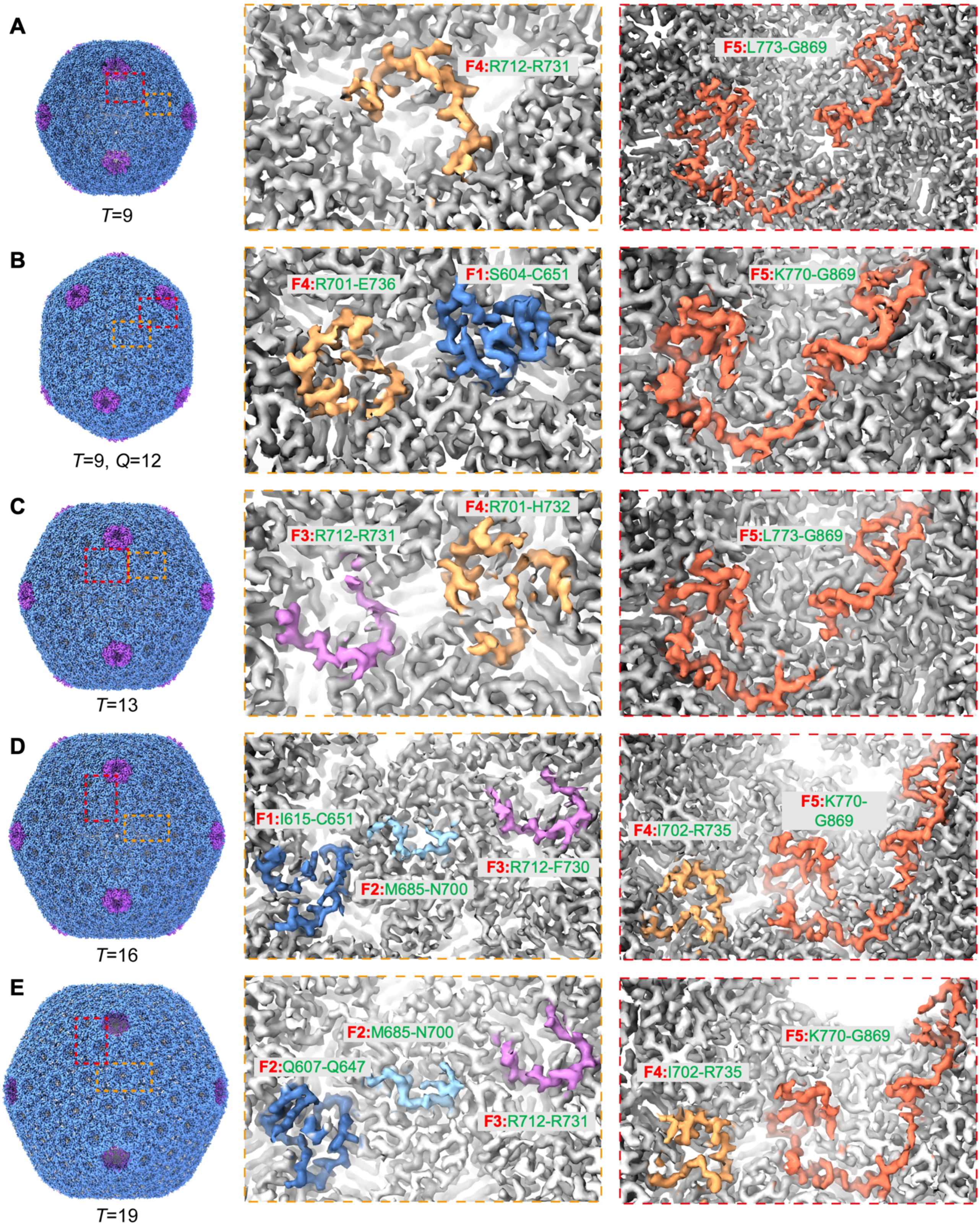

Supplementary Figure 4. CsoS2 density in shell assemblies from the midi-shell construct. (A) *T*=9 shell with a close-up view of CsoS2 F4 and F5 fragments. **(B)** *T*=9 *Q*=12 midi-shell with a close-up view of CsoS2 F1, F4, and F5 fragments. **(C)** *T*=13 midi-shell with a close-up view of CsoS2 F3, F4 and F5 fragments. **(D)** *T*=16 midi-shell with a close-up view of CsoS2 F1-F5 fragments. **(E)** *T*=19 midi-shell with a close-up view of CsoS2 F1-F5 fragments. The F1-F5 fragments are coloured blue, cyan, magenta, yellow, and orange, respectively.

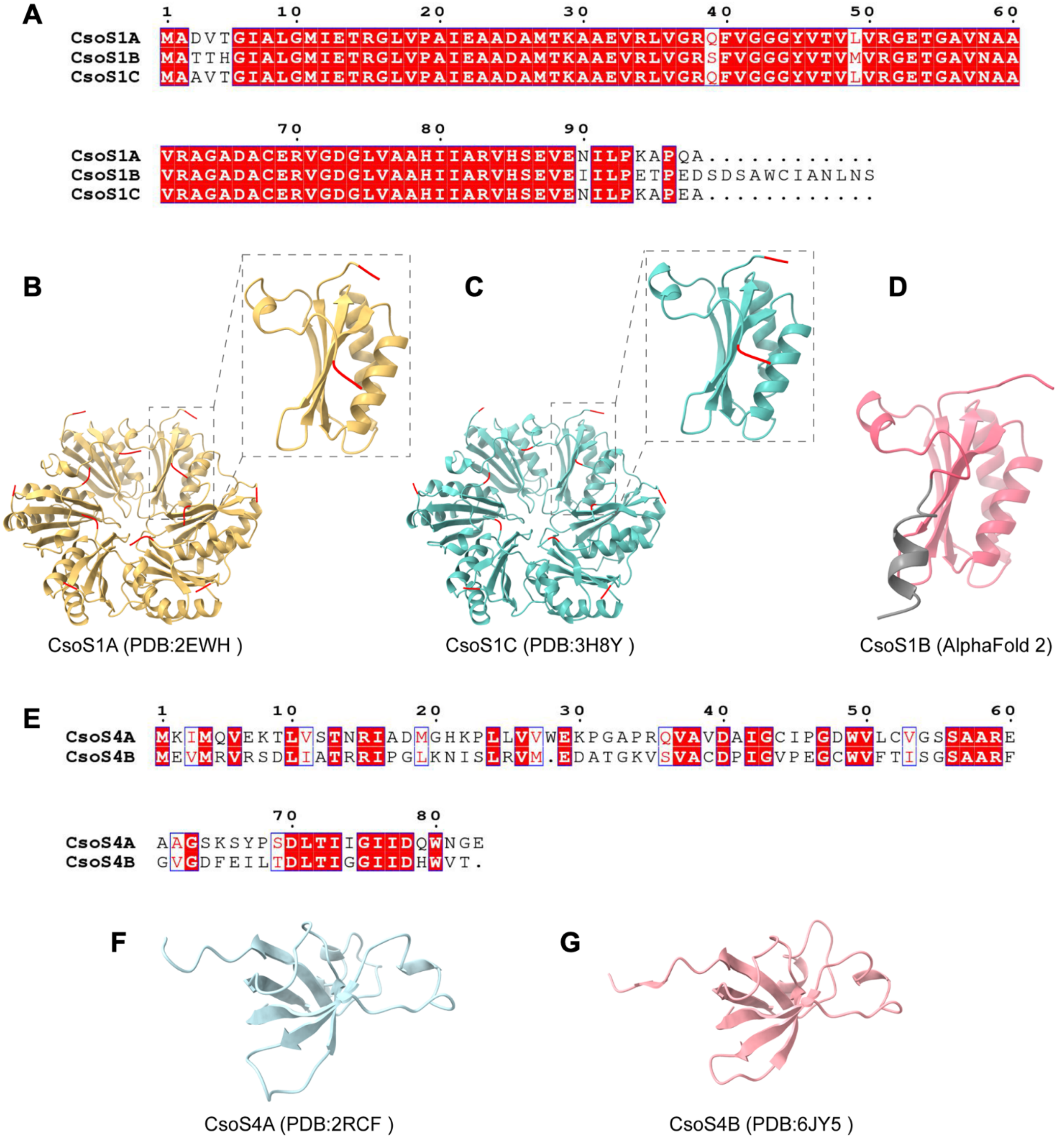

Supplementary Figure 5. Sequence and structural analysis of CsoS1A/B/C and CsoS4A/B. (A) Sequence alignment of CsoS1A, CsoS1B and CsoS1C. **(B-C)** Crystal structure of CsoS1A (B) and CsoS1C (C) in cartoon view. **(D)** AlphaFold2-predicted CsoS1B structure. The C-terminal (99-110) region of CsoS1B is coloured gray. **(E)** Sequence alignment of CsoS4A and CsoS4B. **(F-G)** Crystal structure of CsoS4A (F) and CsoS4C (G).

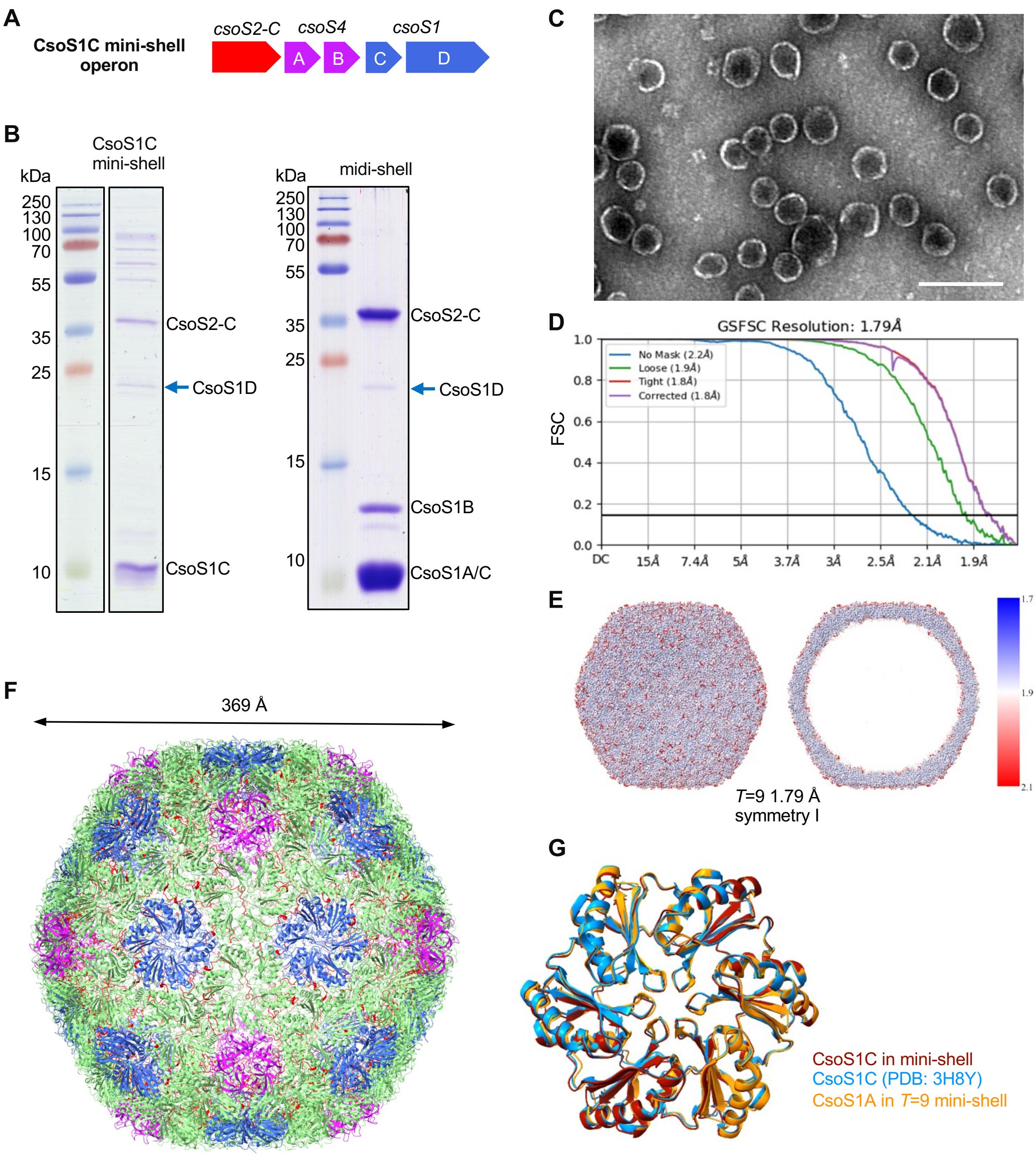

Supplementary Figure 6. Design and overall cryo-EM structure of CsoS1C-formed mini-shells **(A)** Genetic organization of the CsoS1C-containing mini-shell operon. **(B)** SDS-PAGE of purified CsoS1C-formed mini-shells (left) and midi-shells (right). The blue arrows indicate the presence of CsoS1D in both types of shells. The total proteins (15 µg) loaded for midi-shells was 10-fold higher than that for CsoS1C-formed mini-shells to allow the detection of relatively less abundant CsoS1D in midi-shells. **(C)** EM images of negatively stained CsoS1C-formed mini-shells. Scale bar, 100 nm. **(D)** The gold standard Fourier shell correlation (FSC) curves for estimation of the resolution of the density map with a criterion of 0.143. **(E)** Local resolution distributions of the CsoS1C-containing mini-shell cryo-EM map are shown in the top view (left) and central slice (right). **(F)** Cryo-EM structure of the CsoS1C-formed mini-shell with an icosahedral symmetry of *T*=9 at 1.8 Å resolution. The diameter of the shells is ~37 nm. Shell components are coloured purple (CsoS4A pentamer), blue/green (quasi-equivalent CsoS1A hexamer), and red (CsoS2). **(G)** Structural similarity between CsoS1C and CsoS1A in shell assemblies.

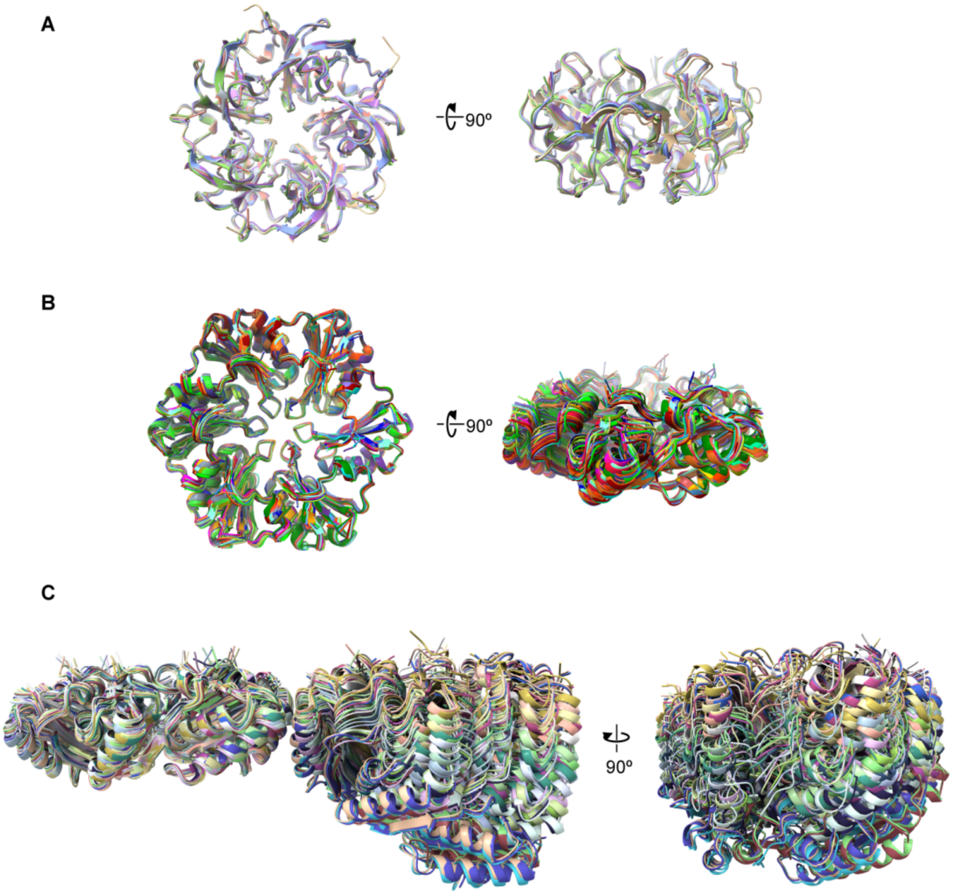

Supplementary Figure 7. Structural comparison of the capsomeres. (A-B) Structural comparison of CsoS4 pentamers (A) and CsoS1 hexamers (B) from *T*=9, *T*=13, *T*=16, *T*=19, and *T*=9 *Q*=12 midi-shell assemblies in two orthogonal views. **(C)** Overlay of the interfaces between hexamer and hexamer (interfaces 2-10) from *T*=9, *T*=13. *T*=16, *T*=19, and *T*=9 *Q*=12 midi-shells viewed from the side. The angles between capsomeres of the hexamer-hexamer interfaces varied in the range of 0-33.6º (Supplementary Table 3). The blocks from different shells and relative positions are in different colours.

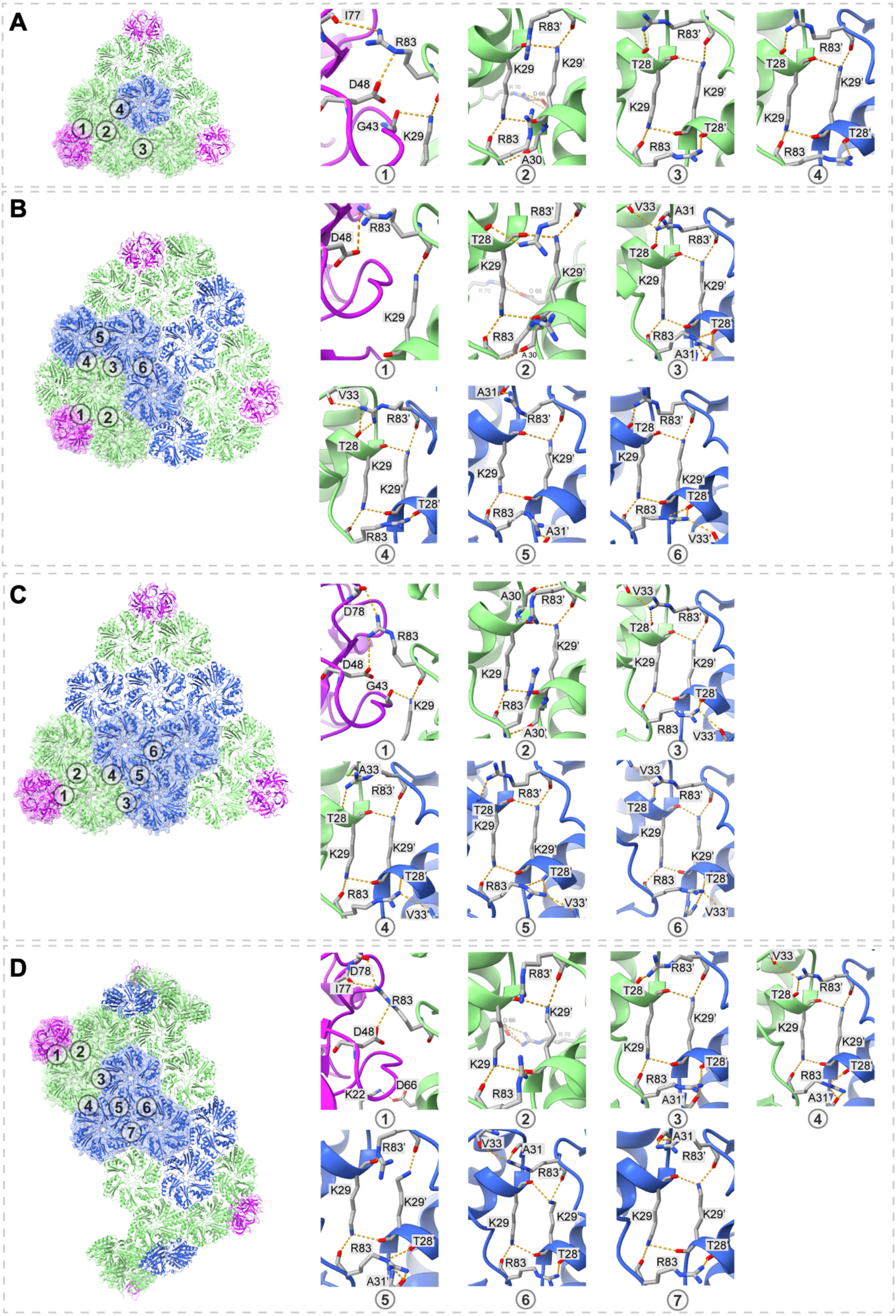

Supplementary Figure 8. The organization in different assembly interfaces of *T*=9 (A), *T*=13 (B), *T*=16 (C), and *T*=9 *Q*=12 (D) shells.

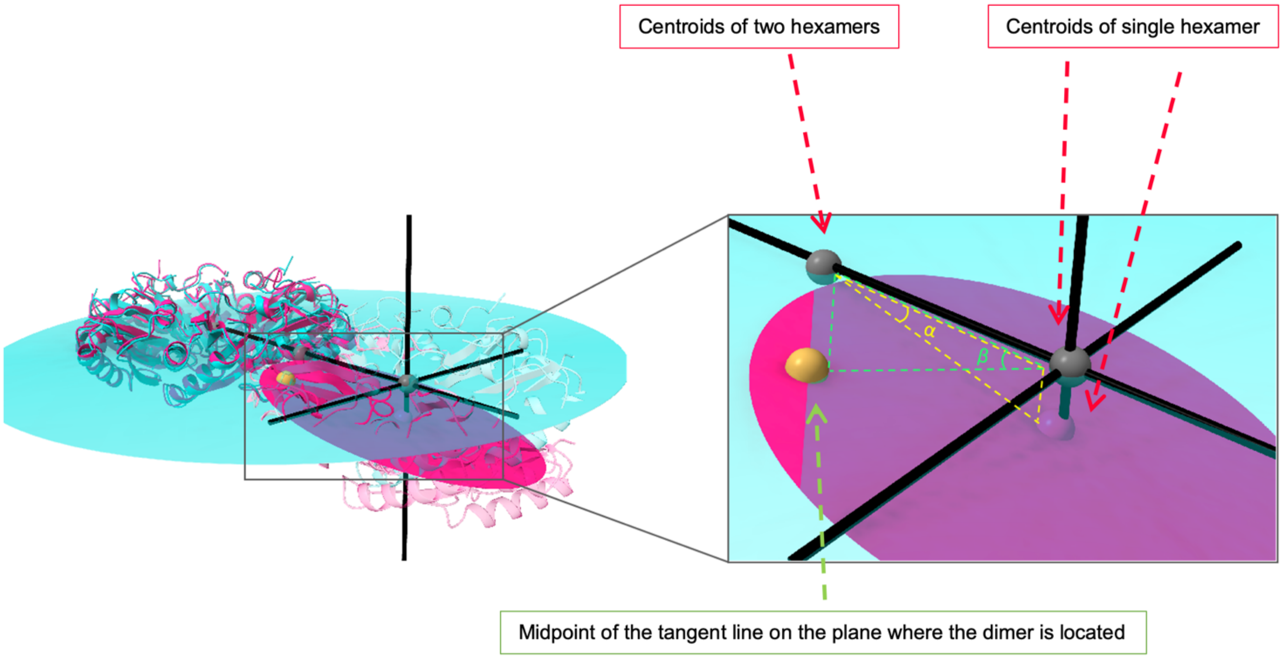

Supplementary Figure 9. Diagrammatic representation of defined α-angle (vertical) and β-angle (horizontal) to differentiate the angular variances between capsomeres in various interfaces.

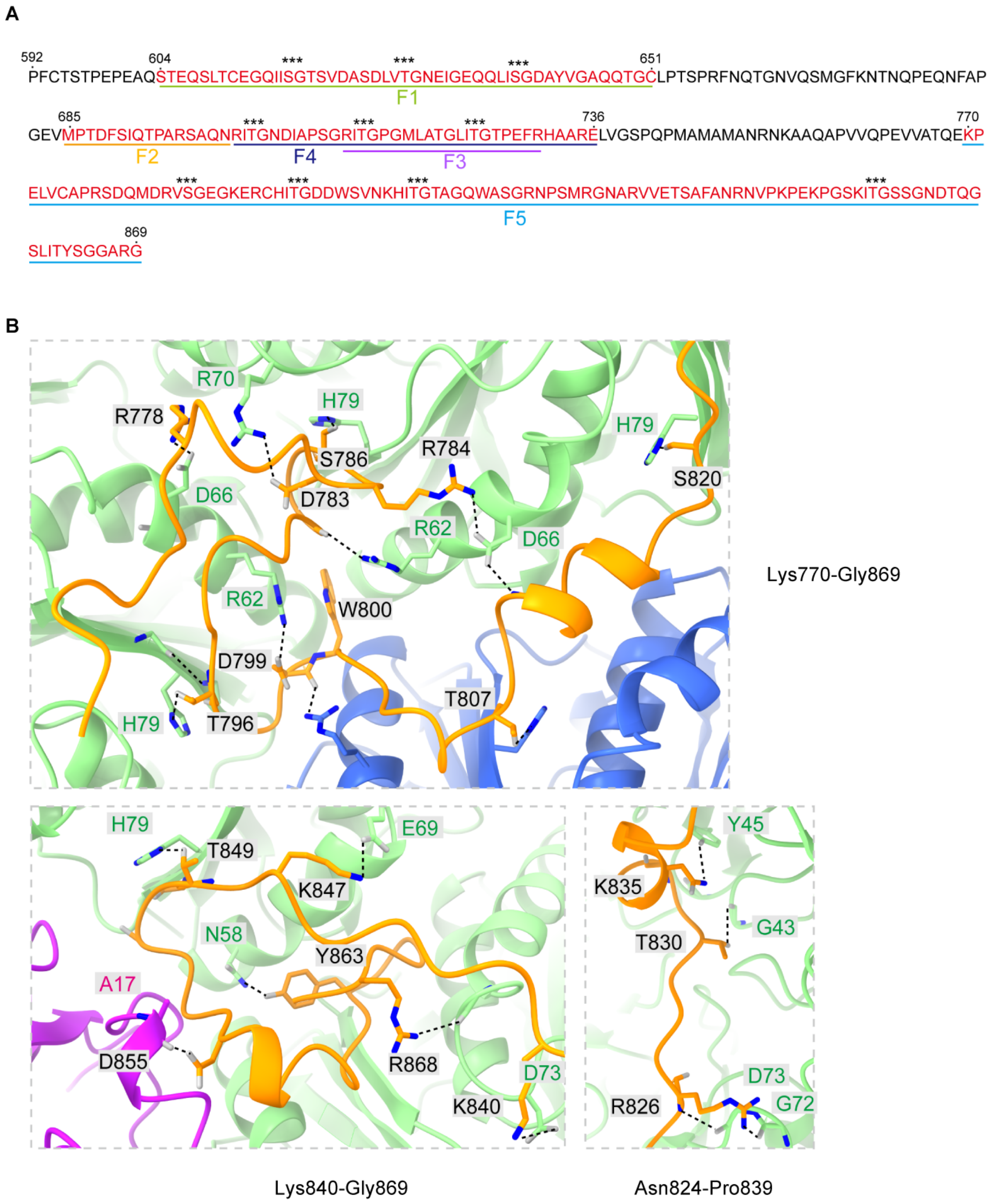

Supplementary Figure 10. Sequence of CsoS2-C and the interaction interfaces of CsoS2-C F5 with the capsomeres. (A) Sequence of CsoS2-C. The five resolved fragments (F1-F5) are underlined in green (F1), yellow (F2), purple (F3), blue (F4), and cyan (F5). The identified sequences in the structures are coloured in red. The [IV][TS]G motifs are marked with asterisks. **(B)** Interaction interfaces of CsoS2-C F5 with the capsomeres. The hexamers adjacent to the pentamer are coloured in green, while those surrounded by other hexamers are coloured in blue. The pentamer is coloured in magenta.

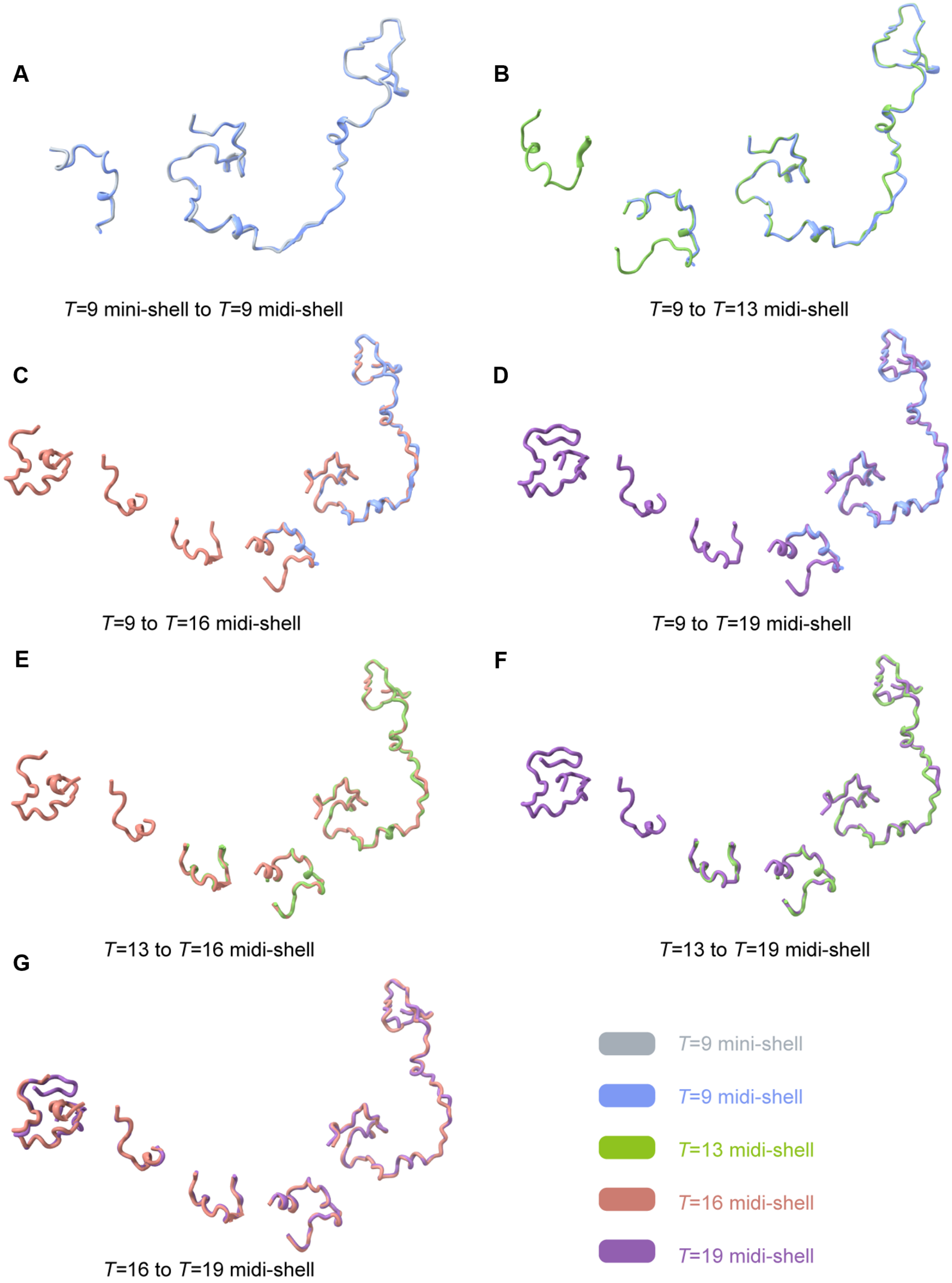

Supplementary Figure 11. Comparison of the identified CsoS2-C fragments from different midi-shells. (A) Comparison of *T*=9 midi-shell and *T*=9 mini-shell. **(B)** Comparison of *T*=9 and *T*=13 midi-shells. **(C)** Comparison of *T*=9 and *T*=16 midi-shells. **(D)** Comparison of *T*=9 and *T*=19 midi-shells. **(E)** Comparison of *T*=13 and *T*=16 midi-shells. **(F)** Comparison of *T*=13 and *T*=19 midi-shells. **(G)** Comparison of *T*=16 and *T*=19 midi-shells. Identified CsoS2-C fragments from *T*=9 mini-shell, *T*=9, *T*=13, *T*=16 and *T*=19 midi-shells are coloured in gray, light blue, lime green, brown, and purple, respectively.

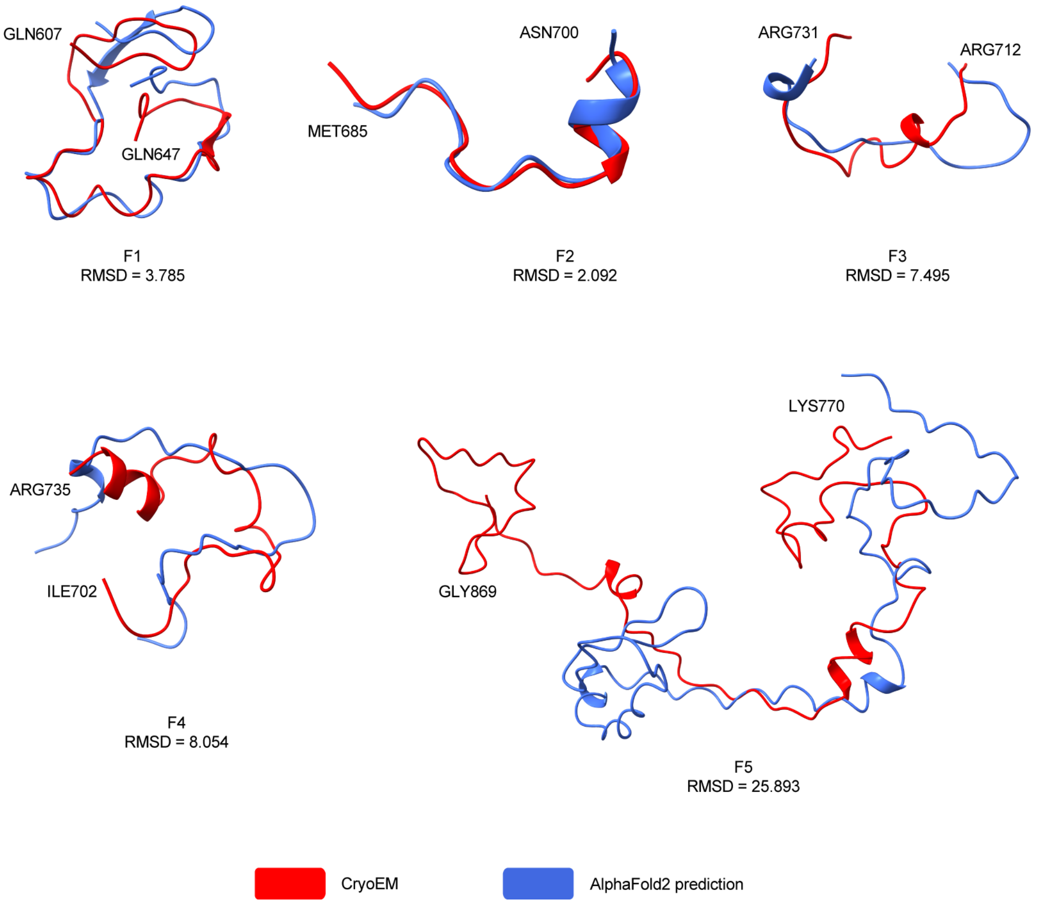

Supplementary Figure 12. Overlay of the CsoS2-C structure from AlphaFold2 prediction (blue) and cryo-EM (red).

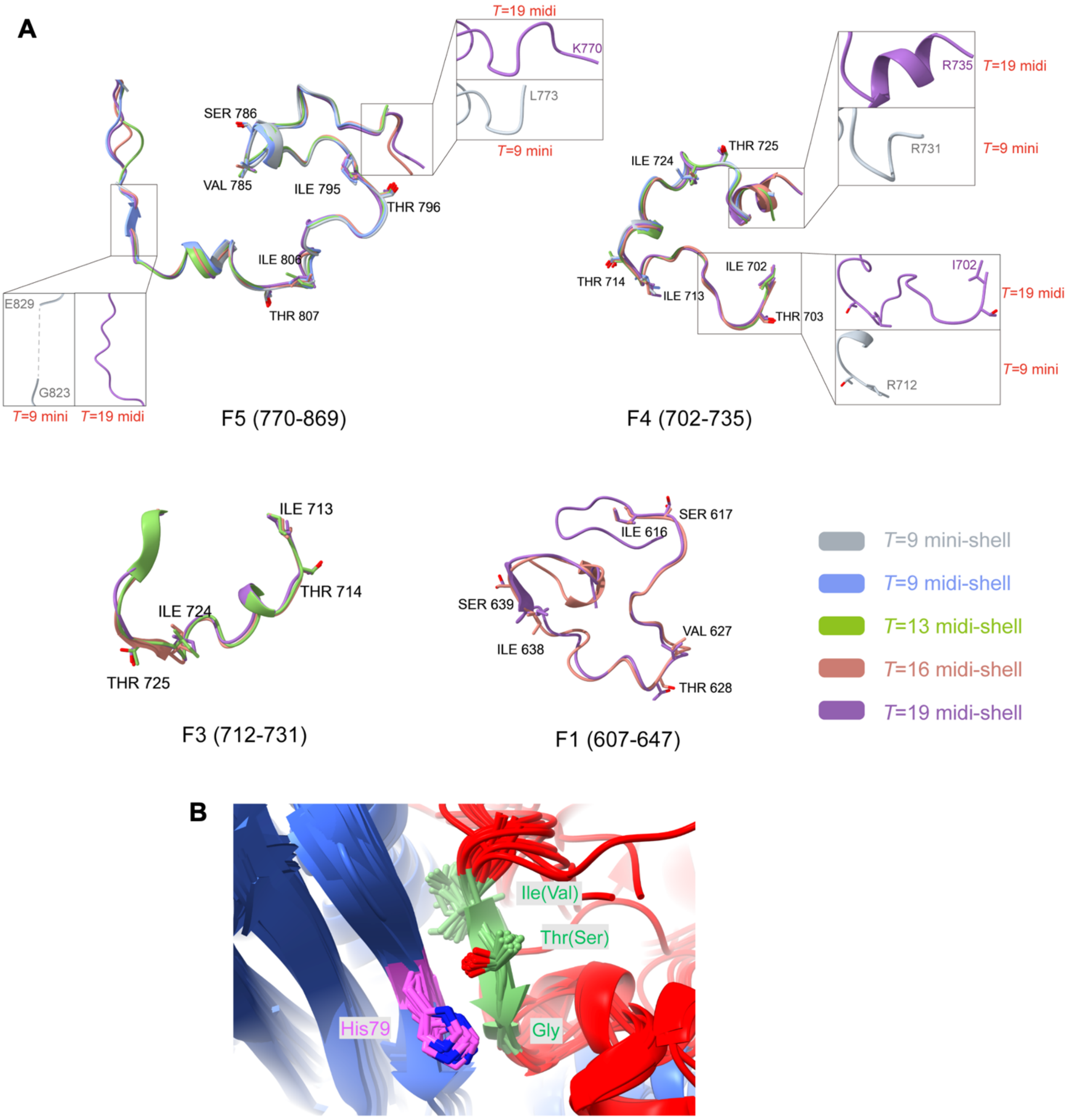

Supplementary Figure 13. Comparison of the identified CsoS2-C fragments from the midi-shells and the *T*=9 mini-shell. (A) Structural alignment of the identified CsoS2-C fragments from *T*=9, *T*=13, *T*=16, and *T*=19 midi-shells. The conservative motifs [IV][TS]G are labeled. Close-up views of the newly identified structures of F4 and F5 in *T*=19 midi-shell compared to *T*=9 mini-shell are shown in the boxes. **(B)** Alignment of [IV][TS]G motifs (green) in CsoS2-C in contact with CsoS1A His79.

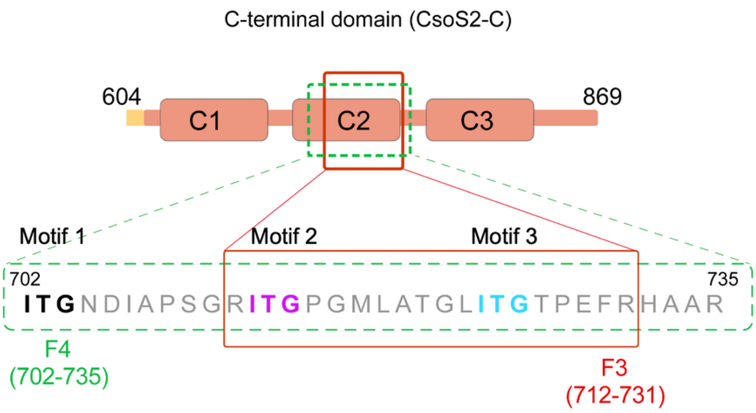

Supplementary Figure 14. The three [IV][TS]G motifs of the Ile702−Arg735 region of CsoS2-C.

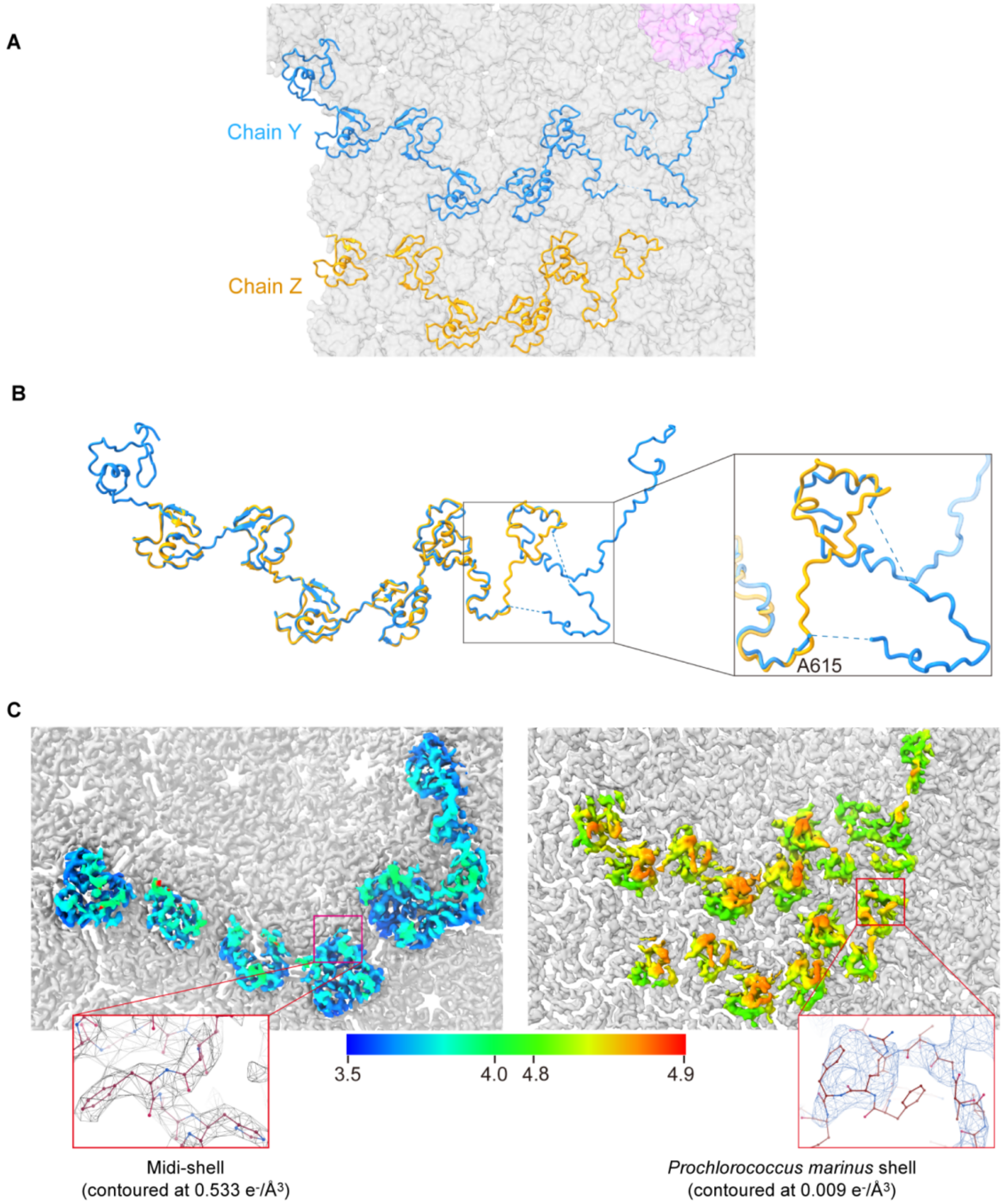

Supplementary Figure 15. Comparison of the identified CsoS2-C between midi-shell (*T*=19) and *Prochlorococcus marinus* MED4 α-carboxysome shell (PDB: 8WXB). (A-B) Structural alignment of the two identified CsoS2 chains, a shorter one (Chain Z, yellow) and a longer one (Chain Y, blue), from *Prochlorococcus marinus* MED4 α-carboxysome shell. A close-up view of the conformational differences between the two chains is shown in the box. **(C)** Comparison of the electron density of CsoS2-C between the data from the *T*=19 midi-shell (left) and the shell of *Prochlorococcus* (right).

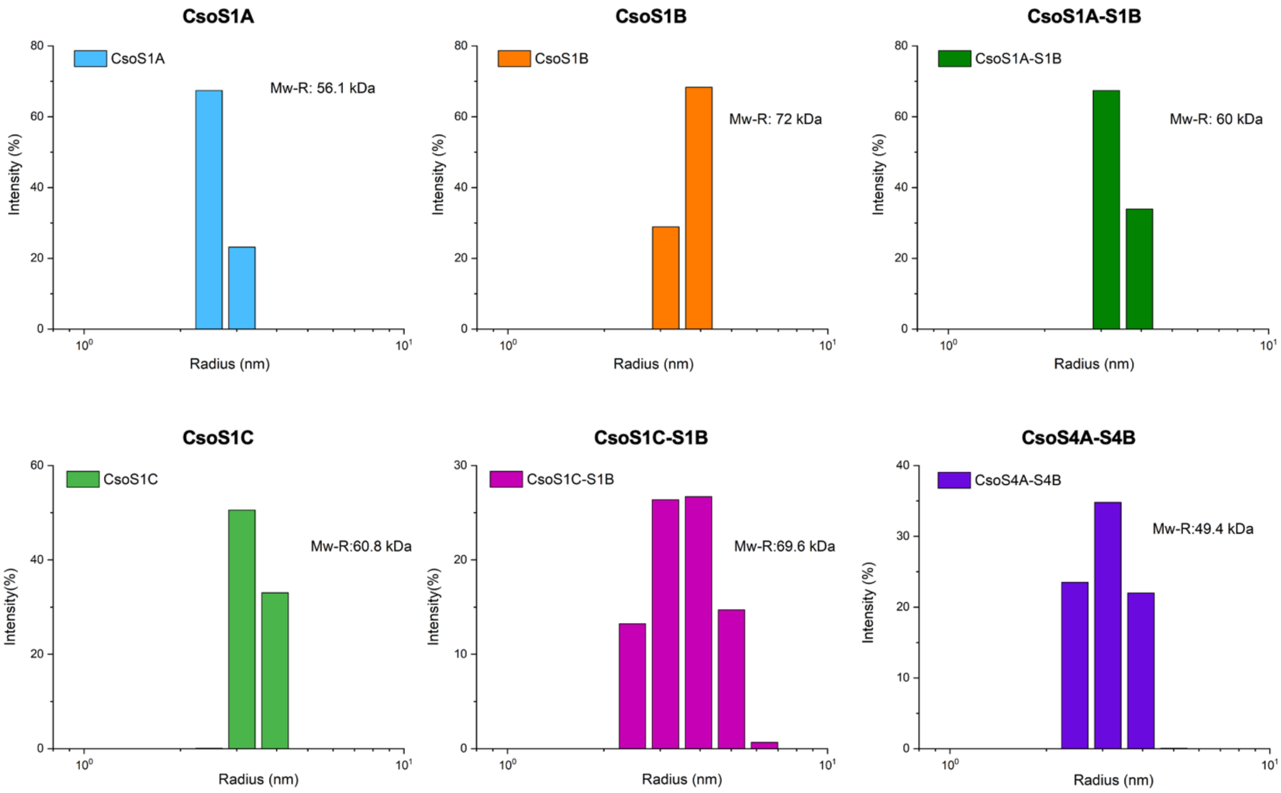

Supplementary Figure 16. Particle size fitting results of dynamic light scattering.

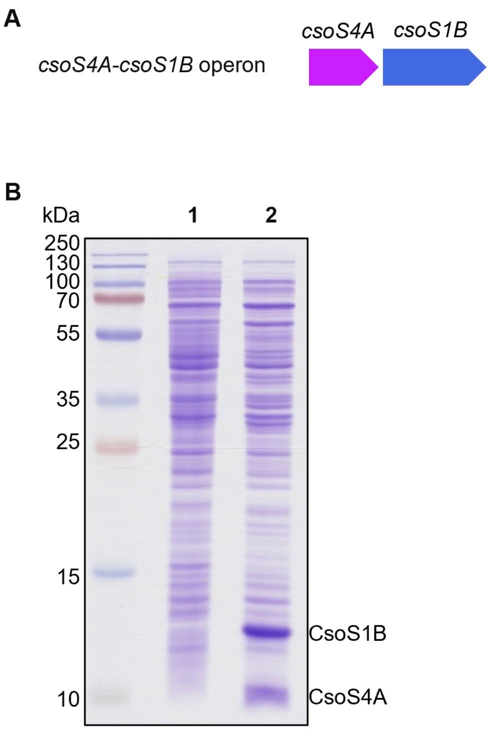

Supplementary Figure 17. Characterization of the CsoS4A-CsoS1B construct. (A) Genetic organization of the *csoS4A-csoS1B* operon. **(B)** SDS-PAGE of cell lysates from *E. coli* cells expressing empty plasmid (1) and the *csoS4A-csoS1B* operon (2), confirming the expression of CsoS1B and CsoS4A from the *csoS4A-csoS1B* operon.

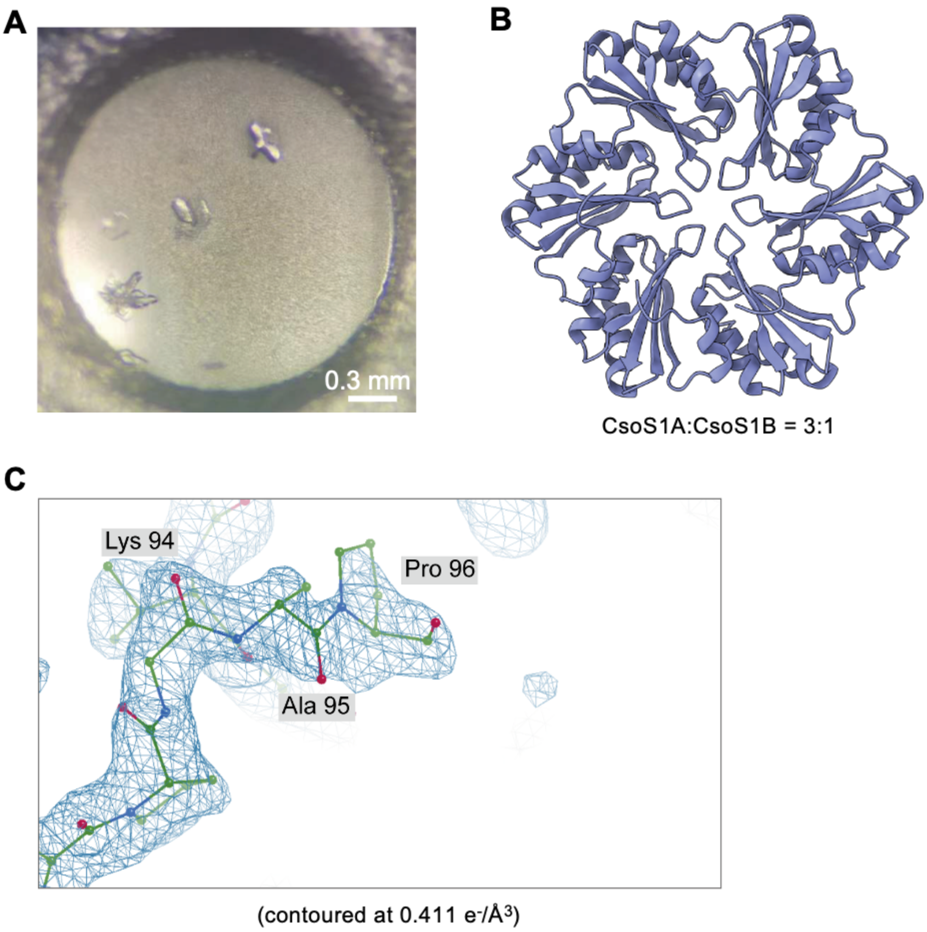

Supplementary Figure 18. Crystals of the CsoS1A-CsoS1B heteromeric hexamer and the modeled structure. (A) The crystal image. **(B)** The modeled structure. Biochemical data confirmed that both CsoS1A and CsoS1B are present in the formed crystals with a ratio of 3:1 (Fig. 4E), suggesting that there are one or two CsoS1B subunits per hexamer on average. However, CsoS1B was unable to be distinguished from CsoS1A in the crystal structure due to data averaging during data processing. Thus, only the sequence of CsoS1A was used in constructing the crystal structure. **(C)** The density map of the C-terminus of CsoS1. In contrast, the densities of the CsoS1B C-terminal α-helix were not observed.

Supplementary Table 1. Cryo-EM data collection, refinement and validation statistics.

	midi-shells							CsoS1C mini-shell
Data collection and processing	*T*=9 (I)	*T*=9 (C1)	*T*=9 *Q*=12 (D5)	*T*=13 (I)	*T*=16 (I)	*T*=16 (C1)	*T*=19 (I)	*T*=9 (I)
Magnification (nominal)	81,000	81,000	81,000	81,000	81,000	81,000	81,000	105,000
Voltage (kV)	300	300	300	300	300	300	300	300
Detector	Gatan K3	Gatan K3	Gatan K3	Gatan K3	Gatan K3	Gatan K3	Gatan K3	Gatan K3
Electron exposure (e^-/Å^2)	50.0	50.0	50.0	50.0	50.0	50.0	50.0	46.1
Defocus range (μm)	-1.2~-2.5	-1.2~-2.5	-1.2~-2.5	-1.2~-2.5	-1.2~-2.5	-1.2~-2.5	-1.2~-2.5	-0.6~-2.0
Pixel size (Å)	0.53	0.53	0.53	0.53	0.53	0.53	0.53	0.829
Symmetry imposed	I	C1	D5	I	I	C1	I	I
Initial particle images (no.)	92,101	92,101	92,101	92,101	92,101	92,101	92,101	1,229,909
Final particle images (no.)	40,177	40,177	3,655	5,953	12,775	12,775	5,119	131,477
FSC threshold	0.143	0.143	0.143	0.143	0.143	0.143	0.143	0.143
Map resolution (Å)	2.30	2.77	2.99	2.93	2.75	3.71	3.04	1.79
Refinement								
Initial model used (PDB code)	8B12	-	8B12	8B12	8B12	-	8B12	8B12
Model resolution (Å)	1.86	-	1.86	1.86	1.86	-	1.86	1.86
FSC threshold	0.143	-	0.143	0.143	0.143	-	0.143	0.143
Model composition								
Non-hydrogen atoms	6719	-	47066	9570	11941	-	13952	6850
Protein residues	935	-	6555	1334	1667	-	1948	932
*B*factor (Å^2)								
Protein	19.20	-	46.23	52.13	37.59	-	53.32	10.78
R.m.s deviations								
Bond lengths (Å)	0.015	-	0.006	0.014	0.016	-	0.011	0.002
Bond angles (°)	1.941	-	1.043	2.361	2.403	-	2.000	0.517
Validation								
MolProbity score	1.09	-	1.18	1.49	1.51	-	1.57	1.03
Clashscore	1.91	-	3.13	2.58	3.19	-	3.54	2.51
Poor rotamers (%)	1.51	-	1.27	3.84	3.33	-	3.01	0.45
Ramanchandran plot								
Favored (%)	99.34	-	98.84	98.77	98.52	-	97.74	99.56
Allowed (%)	0.66	-	1.13	1.23	1.48	-	2.26	0.44
Disallowed (%)	0	-	0.03	0	0	-	0	0
Accession codes	EMD-39598 PDB 8YVE	-	EMD-39599 PDB 8YVF	EMD-39601 PDB 8YVI	EMD-39597 PDB 8YVD	-	EMD-39596 PDB 8YVC	EMD-50109 PDB 9F0H

Supplementary Table 2. Mass spectrometry analysis of purified midi-shells.

Protein	Mass (Da)	Score	Matches[a]	Sequences[b]	Coverage rate
CsoS1C	9970	24265	699(581)	11(11)	95%
CsoS1A	10013	19683	624(525)	11(9)	95%
CsoS1B	11408	12175	420(338)	8(8)	95%
CsoS2-C	29468	1882	65(47)	12(10)	64%
CsoS4A	9000	174	7(3)	2(2)	53%
CsoS4B	8871	96	4(3)	2(2)	41%
CsoS1D	23550	87	5(1)	4(1)	23%

[a] Total number of peptide segments matched, within parentheses are the number of matches above the significance threshold.

[b] Total number of sequences matched, with the number of sequences above the significance threshold in parentheses.

Supplementary Table 3. Statistics of vertical (α) and horizontal (β) angles between pentamer and hexamer (1), and between hexamer and hexamer (2-10) in different interfaces from *T*=9, *T*=13, *T*=16, *T*=19 and *T*=9 *Q*=12 shells.

	***T*=19**		***T*=16**		***T*=13**		***T*=9**		***T*=9 *Q*=12**	
	α	β	α	β	α	β	α	β	α	β
1	29.4°	0°	31.5°	0°	30.4°	0°	30.9°	0°	29.7°	0°
2	28.6°	3.9°	32.8°	0°	33.6°	4.5°	33.2°	0°	33.1°	3°
3	0°	0°	0°	0°	0°	0°	0°	0°	13.4°	13.8°
4	15.1°	38.1°	12.9°	38.8°	13°	46°	19.4°	28.9°	0°	0°
5	13.4°	43.2°	16.5°	29.2°	20.4°	12.4°			20°	17.3°
6	13.6°	13.7°	5.9°	3.7°	10.6°	21.7°			9.6°	42.9°
7	16.2°	12.4°							11.8°	12.2°
8	8.1°	17.8°								
9	4.3°	48.1°								
10	1.5°	70.7°								

Supplementary Table 4. Statistics of major interaction atoms and non-covalent bond length in different interfaces from *T*=9, *T*=13, *T*=16, *T*=19 and *T*=9 *Q*=12 shells.

	***T*=19**		***T*=16**		***T*=13**		***T*=9**		***T*=9 *Q*=12**	
	**Interacting atoms**	**Distances**	**Interacting atoms**	**Distances**	**Interacting atoms**	**Distances**	**Interacting atoms**	**Distances**	**Interacting atoms**	**Distances**
1	D48/OD1&R83'/NH2	3.157 Å	D78/O&R83'/NH1<br>D48/OD1&R83'/NH2	3.169 Å<br>3.717 Å	D48/OD2&R83'/NH1	4.176 Å	I77/O&R83'/NH1<br>D48/O&R83'/NH2	3.522 Å<br>2.844 Å	D78/O&R83'/NH2<br>I77/O&R83'/NH2<br>D48/O&R83'/NH1	2.831 Å<br>3.102 Å<br>4.211 Å
2	H85/NE2&R83'/NH2<br>H85'/NE2&R83/NH1	2.899 Å<br>2.738 Å	K29/O&R83'/NH1<br>A30/O&R83'/N<br>K29'/O&R83/NH1<br>A30'/O&R83/N	2.969 Å<br>3.424 Å<br>3.487 Å<br>3.627 Å	T28/O&R83'/NH2<br>A30'/O&R83/N	3.174 Å<br>3.315 Å	K29/O&R83'/NH2<br>K29'/O&R83/NH1	2.68 Å<br>2.587 Å	K29'/O&R83/NH1	2.496 Å
3	T28/O&R83'/NH1<br>A31/O&R83'/NH1<br>A31'/O&R83/NH2	3.257 Å<br>3.004 Å<br>2.750 Å	T28/O&R83'/NH1<br>V33/O&R83'/NH1<br>T28'/O&R83/NH1<br>V33'/O&R83/NH1	2.911 Å<br>3.269 Å<br>2.422 Å<br>3.515 Å	T28/O&R83'/NH1<br>V33/O&R83'/NH1<br>T28'/O&R83/NH2<br>A31'/O&R83/NH2	2.879 Å<br>3.276 Å<br>3.375 Å<br>3.031 Å	T28/O&R83'/NH1<br>T28'/O&R83/NH1	2.707 Å<br>2.707 Å	T28/O&R83'/NH2<br>T28'/O&R83/NH1<br>A31'/O&R83/NH1	2.716 Å<br>3.109 Å<br>3.061 Å
4	A31/O&R83'/NH2<br>T28'/O&R83'/NH2	3.143 Å<br>3.04 Å	T28/O&R83'/NH2<br>T28'/O&R83/NH2<br>V33'/O&R83/NH2	2.717 Å<br>2.916 Å<br>3.483 Å	T28/O&R83'/NH1<br>V33/O&R83'/NH2<br>T28'/O&R83/NH2	2.277 Å<br>3.472 Å<br>2.454 Å	T28/O&R83'/NH1<br>T28'/O&R83/NH1	2.596 Å<br>2.598 Å	T28/O&R83'/NH1<br>V33/O&R83'/NH1<br>A31'/O&R83/NH2	2.864 Å<br>3.388 Å<br>3.031 Å
5	V33/O&R83'/NH2<br>T28'/O&R83/NH1<br>V33'/O&R83/NH2<br>T28/O&R83'/NH1	3.17 Å<br>2.251 Å<br>3.067 Å<br>3.154 Å	T28/O&R83'/NH1<br>V33'/O&R83/NH2	2.943 Å<br>3.515 Å	A31/O&R83'/NH1<br>A31'/O&R83/NH1	2.777 Å<br>2.777 Å			K29/O&R83'/NH1<br>T28'/O&R83/NH1	3.374 Å<br>2.825 Å

6	A31'/O&R83/NH2<br>V33/O&R83'/NH2	3.442 Å<br>3.491 Å	T28/O&R83'/NH1<br>V33/O&R83'/NH1<br>T28'/O&R83/NH1<br>A31'/O&R83/NH1	2.633 Å<br>3.56 Å<br>2.652 Å<br>3.241 Å	T28/O&R83'/NH1<br>T28'/O&R83/NH2<br>V33'/O&R83/NH2	2.9 Å<br>2.871 Å<br>3.115 Å			A31/O&R83'/NH1<br>V33/O&R83'/NH1<br>T28'/O&R83/NH1	2.573 Å<br>3.504 Å<br>2.705 Å
7	K29/O&R83'/NH2<br>K29'/O&R83/NH2	3.444 Å<br>3.179 Å							A31/O&R83'/NH2<br>T28'/O&R83/NH1	3.175 Å<br>2.58 Å
8	T28/O&R83'/NH2<br>A31/O&R83'/NH2<br>A31'/O&R83/NH2<br>T28'/O&R83/NH1	3.583 Å<br>3.288 Å<br>4.0 Å<br>3.072 Å								
9	T28/O&R83'/NH2<br>A31'/O&R83/NH1	2.701 Å<br>2.371 Å								
10	A31/O&R83'/NH2<br>T28'/O&R83/NH2	3.002 Å<br>2.876 Å								

Supplementary Table 5. Diffraction data and refinement statistics of CsoS1A-CsoS1B heteromeric hexamer.

Parameters	Values
**Data collection**	
Space group	P 63
Unit cell (Å)	a = b = 67.31, c = 64.41
Resolution (Å)	58.29-2.1 (2.16-2.1) [a]
Completeness (%)	99.9 (100) [a]
R_{merge} [b]	0.143 (0.482) [a]
Mean I/sigma(I)	12.8 (5.4) [a]
CC1/2	0.996 (0.99) [a]
**Refinement statistics**	
R_{work} (%)	0.2167 (0.2417) [a]
R_{free} (%)	0.2490 (0.3632) [a]
B-factor (Å^2)	
Protein	31.37
Solvent	36.62
RMSD from ideal geometry	
Length (Å)	0.008
Angles (°)	1.29
Ramachandran plot (%)	
Favored	98.31%
Allowed	1.69%
Accession codes	PDB: 8YXU

[a] Numbers in parentheses refer to data in the highest-resolution shell.
[b] $R_{merge}=\sum_{hkl}\sum_i|I(hkl)_i - <I(hkl)>|/\sum_{hkl}\sum_i<I(hkl)_i>$, where I is the observed intensity, $<I(hkl)>$ represents the average intensity, and $I(hkl)_i$ represents the observed intensity of each unique reflection.

Supplementary Table 6. ssDNA oligonucleotides used in this study. The overlapping sequences for Gibson assembly are underlined.

Primer	Sequence (5'-3')	Description
NcoI-pBAD-S2-C-FW	GGCTAACAGGAGGAATTAACCATGCCGTTTTGTACGAGCACCCCAGAG	Construction of midi-shells
EcoRI-pBAD-S1D-RV	TTGTTCTACGTAAGCTTCG	
EcoRI-FW	CGAAGCTTACGTAGAACAA	
NcoI-RV	CATGGTTAATTCCTCCTGTT	
koS1AB-FW	TGTCTAGTAGGGAAGATGCGC	Construction of CsoS1C mini-shell
ko-S1AB-RV	GCGCATCTTCCCTACTAGACATTAAATAAACTCCTAAGCTGTCACTTTATGC	
pBAD-NcoI-S4A-FW	GGCTAACAGGAGGAATTAACCATGAAAATCATGCAAGTTGAGAAAACG	Construction of *csoS4A-csoS1B* operon
S4A-RV	TTACTCACCATTCCACTGATC	
S4A-S1B-FW	GATCAGTGGAATGGTGAGTAAGTCAGATATTCCTAAGACGG	
pBAD-EcoRI-S1B-RV	TGTTCTACGTAAGCTTCGTTAGCTATTCAGATTTGCGATACACC	
pBAD forward primer	ATTTGCACGGCGTCACACTT	Primers for DNA sequencing
pBAD reverse primer	TCATCCGCCAAAACAGCCAA	
pS1CAB-22b-F	TTTAAGAAGGAGATATACATATGGCAGCAGTAACAGGTAT	Construction of co-expressed CsoS1C-S1B, CsoS4A-S4B, CsoS4A-S4B plasmids
pS1CAB-22b-R	GTGGTGGTGGTGGTGGTGCTCGAGGCTATTCAGATTTGCGATAC	
delS1A-F	CGAAAGCCCCTGAAGCTTAAGTCAGATATTCCTAAGACGG	
delS1A-R	TTAAGCTTCAGGGGCTTTCGGCAGGATGTTTTCGAC	
pS4A-22b-F	AAGAAGGAGATATACATATGATGAAAATCATGCAAGTTGA	
pS4A-22b-R	TGGTGGTGGTGGTGCTCGAGAGTTACCCAGTGATCGA	
pS1A-22b-F	AAGAAGGAGATATACATATGATGGCTGATGTAACTGGTAT	
pS1B-22b-R	TGGTGGTGGTGGTGCTCGAGGCTATTCAGATTTGCGATAC	
